# Benthic Assemblages of the Anton Dohrn Seamount (NE Atlantic): Defining Deep-Sea Biotopes to Support Habitat Mapping and Management Efforts with a Focus on Vulnerable Marine Ecosystems

**DOI:** 10.1371/journal.pone.0124815

**Published:** 2015-05-18

**Authors:** Jaime S. Davies, Heather A. Stewart, Bhavani E. Narayanaswamy, Colin Jacobs, John Spicer, Neil Golding, Kerry L. Howell

**Affiliations:** 1 Marine Biology and Ecology Research Centre, Plymouth University, Plymouth, PL4 8AA, United Kingdom; 2 British Geological Survey, Murchison House, West Mains Road, Edinburgh, EH9 3LA, United Kingdom; 3 Scottish Association for Marine Science, Scottish Marine Institute, Oban, Argyll, PA37 1QA, United Kingdom; 4 National Oceanography Centre—Southampton, European Way, Southampton, Hampshire, SO14 3ZH, United Kingdom; 5 Joint Nature Conservation Committee, Monkstone House, City Road, Peterborough, PE1 1JY, United Kingdom; Bangor University, UNITED KINGDOM

## Abstract

In 2009 the NW and SE flanks of Anton Dohrn Seamount were surveyed using multibeam echosounder and video ground-truthing to characterise megabenthic biological assemblages (biotopes) and assess those which clearly adhere to the definition of Vulnerable Marine Ecosystems, for use in habitat mapping. A combination of multivariate analysis of still imagery and video ground-truthing defined 13 comprehensive descriptions of biotopes that function as mapping units in an applied context. The data reveals that the NW and SE sides of Anton Dohrn Seamount (ADS) are topographically complex and harbour diverse biological assemblages, some of which agree with current definitions of ‘listed’ habitats of conservation concern. Ten of these biotopes could easily be considered Vulnerable Marine Ecosystems; three coral gardens, four cold-water coral reefs, two xenophyophore communities and one sponge dominated community, with remaining biotopes requiring more detailed assessment. Coral gardens were only found on positive geomorphic features, namely parasitic cones and radial ridges, found both sides of the seamount over a depth of 1311–1740 m. Two cold-water coral reefs (equivalent to summit reef) were mapped on the NW side of the seamount; *Lophelia pertusa* reef associated with the cliff top mounds at a depth of 747–791 m and *Solenosmilia variabilis* reef on a radial ridge at a depth of 1318-1351 m. Xenophyophore communities were mapped from both sides of the seamount at a depth of 1099–1770 m and were either associated with geomorphic features or were in close proximity (< 100 m) to them. The sponge dominated community was found on the steep escarpment either side of the seamount over at a depth of 854-1345 m. Multivariate diversity revealed the xenophyophore biotopes to be the least diverse, and a hard substratum biotope characterised by serpulids and the sessile holothurian, *Psolus squamatus*, as the most diverse.

## Introduction

Increasing pressure on the marine realm has resulted in the need for greater understanding and better spatial management of our marine environment. This is happening at both national and global levels and in particular there is a real impetus for the establishment of networks of Marine Protected Areas (MPAs) driven by global, European and national (within the UK) initiatives [1–3]. One of the criteria by which MPAs are selected includes the protection of habitats and species that have been identified as rare, sensitive, functionally important, threatened and/or declining [4].

A number of habitats are listed under these various initiatives including, but not limited to, cold-water coral reefs, coral gardens, sponge dominated communities, and communities that are composed of epifauna that provide a structural habitat (e.g. xenophyophores and sea pens) for other associated species. These habitats are listed in the Annex of the International Guidelines for the Management of Deep-Sea Fisheries in the High Seas [5] as examples of species groups, communities, habitats and features that often display characteristics consistent with Vulnerable Marine Ecosystems (VMEs). OSPAR has highlighted a number of deep-sea habitats as ‘threatened or declining’, and these include amongst others, seamounts, *Lophelia pertusa* reefs and coral gardens; while cold-water coral reefs, coral gardens and sponge dominated communities are defined as Annex I listed ‘reef’ habitat under the Habitat Directive [1].

Seamounts are large topographical features often characterised by complex hydrodynamic regimes [6]. They exert an influence over ocean currents by interrupting the flow of water, this can cause tides to be amplified creating fast currents and producing eddies [7, 8]. These elevated currents have a functional role by increasing local food supply, erosion and deposition of sediment, and in some cases exposing hard substratum for faunal attachment thereby increasing larval supply and species recruitment; thus making seamounts diverse biological features [9].

Seamounts support a rich fauna, and are considered biodiversity hotspots with high levels of endemism [6, 9, 10, 11, 12, 13, 14] thus providing a significant role in species dispersal by acting as stepping stones [9, 10, 11]. The increased species richness (as compared to the surrounding seafloor) is suggested to be a result of high carbon inputs to these systems and the availability of habitats [13]. In recent years seamount ecology and hydrography have received considerable research interest in an attempt to understand seamount ecosystems [13, 15, 16, 17, 18].

To date, a small number of MPAs have been established in the NE Atlantic deep-sea/High Seas using one or more of these legal mechanisms. Existing MPAs have been designated based on the known occurrence of *Lophelia pertusa* e.g. NEAFC/EU fisheries closures and UK Government proposals for Sites of Community Importance on Hatton and Rockall Banks [19–21], or are seamounts e.g. NEAFC fisheries closures on the Mid Atlantic Ridge (MAR) [22]. The NEAFC fisheries closures on the MAR fall within a proposed OSPAR MPA [23] which encompasses a large area north and south of the Charlie-Gibbs Fracture Zone. To date there have been no closures made for other listed habitats even though seamounts are listed under Annex V of OSPAR as features that may support VMEs [5]. Seamounts provide important habitats and harbour many listed species (e.g. *Lophelia pertusa*), and as such need protection through the inclusion into MPAs. Anton Dohrn Seamount is just one of several seamounts found in the NE Atlantic that is known to support VMEs.

One of the principal difficulties in designating MPAs for the protection of listed deep-sea habitats is a lack of detailed distribution data. Mapping these habitats is vital for policy makers and governments if their offshore areas are to be managed properly. Habitat mapping can be undertaken at different spatial scales, ranging from individual organisms up to ecosystems and landscapes [24]. The use of large topographical features such as seamounts and banks (megahabitat landscape features, *sensu* Greene *et al*. [25]) at scales of kilometres to tens of kilometres has been used [26], and proven useful for broad scale mapping over large areas. Whilst broad scale mapping may adequately represent some habitats, others are not distributed at the same spatial scale and thus require a different approach. To adequately represent the biology it is necessary to understand the distribution of habitats at a data acquisition level, or fine-scale, which can be related to typically more generalised, broad scale maps that cover a wider geographic area.

A necessary prelude to mapping is identifying and describing the biological assemblages (biotopes). Biological assemblages or ‘biotopes’ are used as mapping units, where they represent distinct biological assemblages associated with certain environmental factors such as substratum and depth [27]. Existing deep-sea biotopes are generally defined on the basic of the epibenthic megafauna, which are most sensitive to anthropogenic disturbance, thus changes in this community can be an easily monitored indictor for small compartments [28]. Additionally megafauna are known to increase structural complexity and provide shelter for other organisms, thus increasing local diversity [29]. To allow comparability between maps from different areas/regions, essential for the implementation and management of MPAs, it is important to use consistent mapping units and to have adequate descriptions for these habitats so that these terms can be used across geographic regions [25, 30]. In the deep sea there have been few attempts to produce descriptions of benthic assemblages for use in mapping [31–37]. Whilst some benthic assemblages are broadly recognised through the scientific literature, e.g. cold-water coral reefs and ostur (sponge communities), others have been described through the political process (e.g. coral gardens [38]). Few listed deep-sea habitats are supported by scientifically robust descriptions of community composition such that coherent mapping units can be described, and the relationship between listed habitats and ‘more easily mapped’ geomorphological features remains unknown.

Relationships between biological assemblages and geomorphological features at a mesohabitat scale (tens of metres to a kilometre; *sensu* Greene *et al*. [25]) have been reported, e.g. cold-water coral reefs associated with mound features [34, 39–42]. Once the relationship between biological assemblages and geomorphology is understood, it may then be possible to relate biological assemblages to broad-scale geomorphological features which are inherently more easily mapped and can be used as a surrogate to predict the distribution of benthic habitats. This approach is particularly important in the deep sea and for habitats of conservation concern such as listed habitats.

The aims of this paper are to 1) identify the deep-sea megabenthic assemblages of Anton Dohrn Seamount that can be used as classification units in habitat mapping efforts, 2) identify biotopes of conservation interest under international/national policy and 3) to describe the distribution of these biotopes in relation to the geomorphology of the seamount system.

## Methods

### Ethics statement

No specific permissions were required for these locations (57.28°N 10.82°W—57.61°N 11.22°W) and the field studies did not involve collection of endangered or protected species.

### Study area

**Fig 1:**
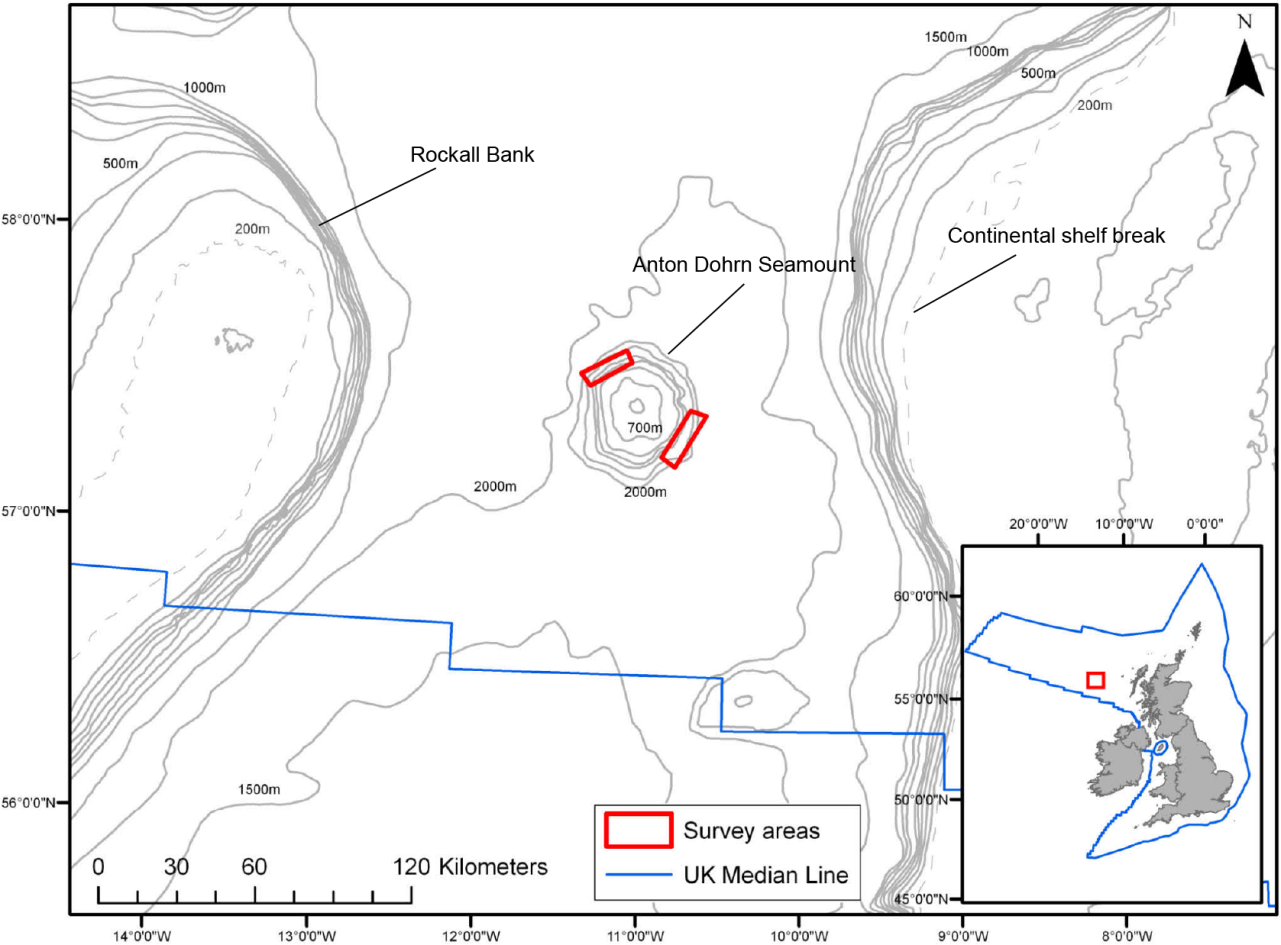
The study area west of Scotland, UK. Survey areas on the NW and SE sides of Anton Dohrn Seamount are marked. Bathymetric contours are provided by GEBCO, the 200 m depth contour (dashed line) marks the approximate position of the continental shelf break.

Anton Dohrn Seamount is a roughly circular seamount centred at 57° 27’ N, 11° 5’ W, located to the west of Scotland in the Rockall Trough between the Hebrides Shelf and Rockall Bank (Fig 1). This steep sided seamount is approximately 45 km in diameter with a relatively flat summit at a depth of ~600 m below sea level rising from a depth of over 2000 m. A number of pinnacles are located on the summit of the seamount, the highest of which reach 530 m below sea level. Anton Dohrn Seamount is also encircled by a well-developed moat feature which deepens from the north-west (~2195 m) to the south-east (~2300 m). The bulk of the seamount probably comprises basaltic lavas [43–45] with a south-east thickening wedge of sediments on the top of the seamount identified on British Geological Survey 2D seismic reflection data.

The hydrographic activity within the Rockall Trough is complex and has been studied for many years. Eastern North Atlantic Water flows along the continental slope and enters the southern part of the Rockall Trough and circulates in an anti-cyclonic direction and exits the trough to the north-west [46]. The slope current continues to transport warm, saline water along the shelf northwards across the Wyville-Thomson Ridge into the Faroe-Shetland Channel [47]. The north-west flank of Anton Dohrn Seamount may be influenced by Arctic waters that periodically overflow the Wyville-Thomson Ridge but it is unlikely that the SE side of the seamount is influenced by this current [48].

### Sampling methods

In 2009, the north-west and south-east flanks of Anton Dohrn Seamount were surveyed using multibeam echosounder and video ground-truthing to identify VMEs (Fig 1) over a four week period (July 2009) using the commercial research vessel *MV Franklin*. High resolution acoustic data were collected using a hull mounted Kongsberg EM710 multibeam echosounder system capable of operating in water depths up to around 2000 m. Swath width was between 1.0–1.5 km and the operating frequency range for the system was 70–100 kHz. Data were processed on-board and gridded at a resolution of 15 m to allow detailed interpretation of meso-scale geomorphological features.

In order to characterise the megabenthic assemblages of the seamount, transects were selected to capture the range of habitats/terrains on either side of the seamount, with a particular focus on topographical features such as mounds. This was achieved by using the processed multibeam bathymetry and backscatter intensity data to select transects that represented different depths, interpreted geomorphology and seabed substratum (inferred from backscatter intensity data). Where time permitted and adequate number of targets were present (e.g. depth range, geomorphology and seabed substratum) replicate transects were undertaken within and between the two study areas. The orientation of the transects reflect the topography, orientation of the geomorphological feature, to maximise the depth range sampled, or was determined by the prevalent oceanic currents and weather conditions that impact safe deployment and recovery of equipment.

**Fig 2:**
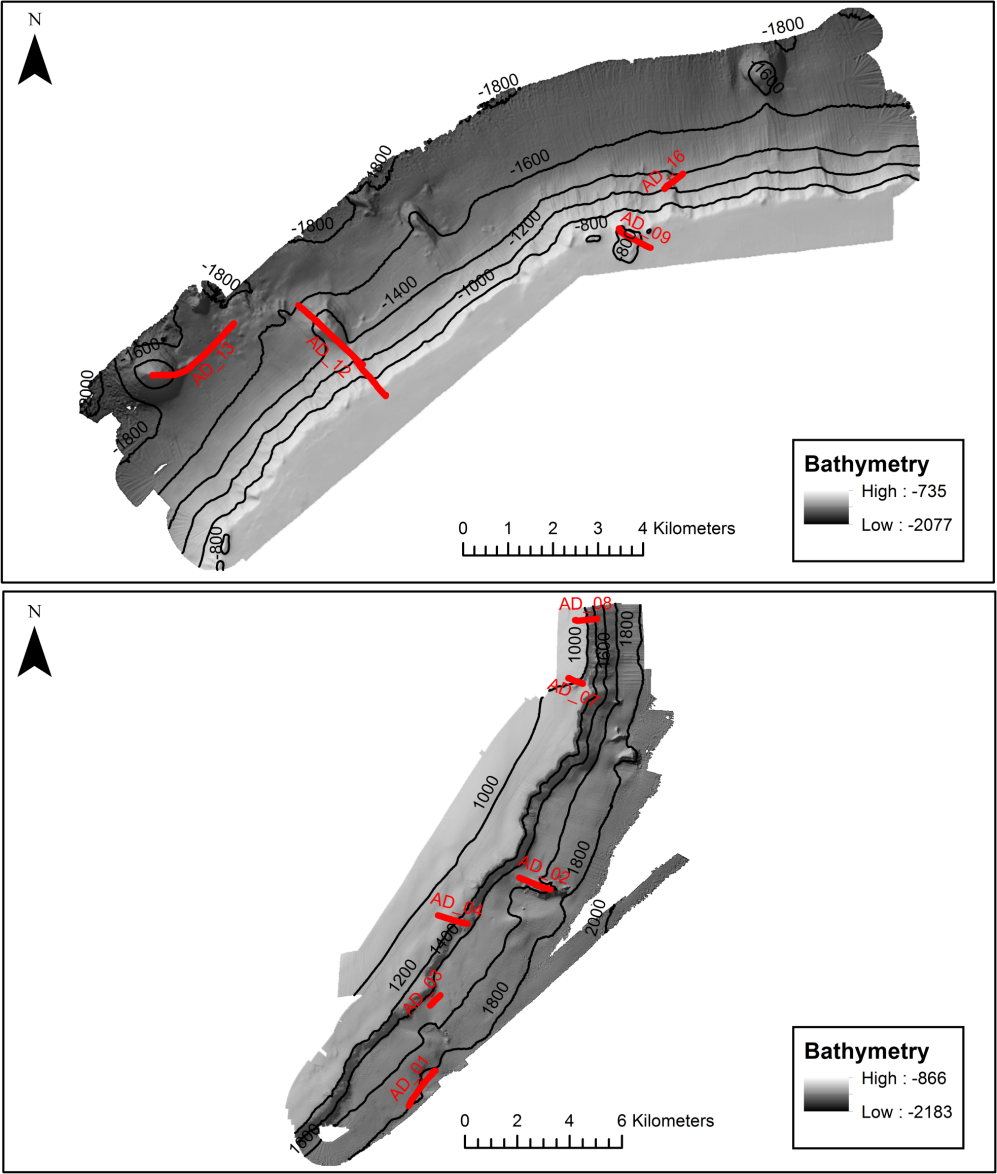
Camera transects sampled from the NW (top) and SE (bottom) survey areas of Anton Dohrn Seamount overlaid on multibeam bathymetry. Selected bathymetric contours are shown as dashed lines with labelled water depths in metres below sea level.

Video and image data were collected from a total of ten transects (four located in the north-west and six in the south-east; Fig 2.) using a drop-frame camera system towed 1–3 m above the seabed at a vessel speed of approximately 0.5 kn. As targeted featured varied in length, so did the length of transects to allow complete sampling of the features. The system comprised a 5 megapixel Kongsberg OE14-208 digital stills camera and an integrated DTS 6000 digital video telemetry system. The drop-frame is also fitted with sensors to record depth, altitude above the seabed and temperature, and an ultra-short baseline (USBL) beacon (calibrated before use), which is fully integrated with the vessel’s digital geographic positioning system (DGPS), to collect accurate positional data for the camera frame [49]. Following the MESH guidelines for data collection [50], a 2–5 minute camera stabilisation period was undertaken at the beginning of each transect to ensure the camera was moving at a constant speed. Video footage was recorded along the entire transect, and at approximately one minute intervals the drop-frame was landed and a stills image taken (sampling unit) hereinafter referred to as a ‘sample’ image. A calibration grid, of known dimensions, was attached to the frame prior to initiation of sampling to determine image area when the frame was on the seabed (0.156 m^2^), 1 m (0.539 m^2^), 2 m (2.11 m^2^) and 3 m (3.65 m^2^) off the seabed and related to sample image to allow abundance/percentage area cover of fauna to be expressed. Transect length varied between 0.5 km and 3.3 km over a depth range of 747–1887 m. Variation in length was due to transects targeting specific features which varied in size, impact of oceanic currents and adverse weather during operations.

### Biological analysis

#### Quantitative analysis of image data

The sample images were quantitatively analysed using the calibration grid as a measure of area. Identification of species from images is often difficult; however observed organisms were identified as distinct morphospecies (morphotypes). All visible organisms >1 cm (at widest point) were identified as distinct morphospecies and assigned an Operational Taxonomic Unit (OTU) number [51]. OTUs were identified to the lowest possible taxonomic level. All individuals were counted except in the case of encrusting, colonial and lobose forms, where percentage cover of the organism was recorded. Highly mobile species such as fish were removed prior to data analysis. Image data were expressed as either individuals/1 m^2^ or percent cover/1 m^2^. In accordance with JNCC guidelines, data were archived with the MEDIN Data Archive Centres http://www.oceannet.org/.

#### Community analysis

Standard multivariate community analysis techniques [52] were used to identify faunally distinct benthic assemblages within the study area. Standardisation of count and cover matrices was undertaken to place them on a common scale to allow a single combined analysis (R. Clarke *pers*.*comm*; [34]). Each matrix was square root transformed, divided through itself and multiplied to put on a common scale of 0.005–0.75 (count * 200 and cover *100). Cluster analysis with group-averaged linkage was performed using a Bray-Curtis similarity matrix derived from transformed, combined species count and percent cover data. The SIMPROF routine was used to identify significant clusters using a significance level of p < 0.01 and the SIMPER routine used to identify those species that characterised significant clusters. Characterising species were defined as those species with a high sim/SD ratio [53], and contributed > 5% to that cluster’s similarity.

### Characterising mapping units (biotopes)

There is a discrepancy between the faunal assemblages identified using community analysis methods and what is required from a practically applicable mapping unit used in producing necessary generalised maps of variation in the biological composition of the seabed. To characterise practical mapping units that can be mapped at a scale appropriate to that of the acoustic data, those clusters identified as faunally distinct using standard cluster analysis techniques were assessed against a second set of criteria to determine their use as mapping units. Only those clusters which met these criteria were further analysed in terms of their faunal composition and diversity. To function as a mapping unit assemblages must, 1) occur at a scale relevant to the resolution of the acoustic data, and the scale of existing, widely accepted benthic communities such as cold-water coral reefs (10 m scale), and 2) be easily identified from video data.

Biotopes were defined in terms of their characterising species, as determined by SIMPER analysis, together with the range of environmental conditions over which they occurred in this study. A One-way Analysis of Similarity (ANOSIM) was undertaken on a normalised, Euclidean distance matrix of environment data (depth and temperature) to test if environmental conditions were different between biotopes.

To assess whether biotopes could be considered of conservation concern, identified biotopes were compared with current definitions of OSPAR and the EC Habitats Directive listed habitats. In identifying those that would be considered as VMEs we used the Annex to the guidelines of the FAO [5] and current OSPAR definitions. This limits the identification of VMEs to those biotopes that contain the example species or communities identified in that Annex. We have not considered biotopes according to the wider criteria listed in the full document.

#### Diversity indices

Megafaunal diversity in terms of species richness and dominance were measured to compliment the characterisation of biotopes, and allow a more complete description of assemblages. Simpson’s Reciprocal Index [1/D] was measured using the DIVERSE routine in Primer v6 [52] to give Simpson’s diversity index (λ) and the reciprocal form taken by 1/D. Simpson’s Reciprocal index was chosen as a measure of dominance as it is less sensitive to sample size [54]. Count and cover data were measured separately for each sample image and then averaged to give a single Simpson measure per image, and expressed as the mean Simpson’s Reciprocal Index per biotope.

Species richness was measured using three methods: 1) mean species richness per biotope, 2) rarefaction, 3) incidence-based species richness estimators (ICE, Chao 2, Jackknife 1 and 2, and bootstrap) using EstimateS 8.3. Rarefaction curves estimate expected species richness (Mao Tau Sobs) for a sub-sample of the pooled total species richness, based on the species actually discovered (as opposed to estimators that estimate species richness including species not sampled) [55] and allow interpolation at lower sample size, thus overcoming sampling bias and varying sample size [56]. Jackknife is a non-parametric species richness estimator which removes subsets of the data and re-calculates the estimator using the reduced samples, and is a good technique for reducing bias [55, 57]. There are two Jacknife estimators: First order Jackknife is a function of the number of rare species in a community, where it calculates the number of species that occur in only one sample; and second order Jackknife which additionally calculates the number of species that occur only in two samples in a community [58]. ICE (incidence-based estimator) estimates the sample coverage, by the proportion of assemblage richness represented by the set of replicated incidence samples. That is, the proportion of all frequencies of infrequent species (found in 10 or fewer samples) which are not unique species. Chao 2 is an asymptote estimator of minimum richness [55]. Bootstrap is a resampling procedure.

A single diversity index is often not sufficient to allow adequate comparisons between assemblages [59] thus multiple indices were used to compare diversity of biotopes. ANOSIM tests (Primer v6 [52]) on Euclidean distance resemblance matrices were undertaken to test for significant differences in diversity between biotopes (H^o^: no significant difference in diversity between biotope). A suite of normalised diversity measures [Simpson’s Reciprocal Index, expected species richness (Sobs) and the five incidence-based estimators] were used to give a holistic view of the diversity measure.

#### Distribution of biotopes in relation to meso-scale geomorphology

Geomorphological classes were interpreted from the multibeam echosounder data (Table 1). Video transects were reviewed and visually classified (guided by the sample image analysis cluster output) using the newly defined biotopes, and changes of biotope type within a transect mapped using ArcGIS 9.3. Biotope mapped video data were overlaid on an interpreted geomorphology polygon layer in ArcGIS and used to qualitatively describe the distribution of biotopes in relation to meso-scale geomorphology, particularly focusing on those biotopes identified as VMEs. Abiotic data were also extracted from the mapped data to define the environmental range of the distribution of each biotope.

**Table 1:** Meso-scale geomorphological features identified from the summit and flank of Anton Dohrn Seamount.

Summit	Flank
Cliff-top mounds	Flute
Cliff edge	Escarpment
	Parasitic cone
	Radial ridge
	Landslide/Rockfall
	Furrow/moat

## Results

### Biological data

Over 14 hours of video and 2745 stills images were collected, of which 744 images were designated sample images. Forty-three sample images were omitted from the analysis due to poor quality, and 41 samples that captured abrupt changes in substratum were added to the analysis. Due to the taxonomic complexity of the images, time constraints did not allow for analysis of all sample images, thus every third sample (*approx*. 3 min intervals) was quantitatively analysed (320 images). On the NW survey area transects were collected over a depth range of 747–1770 m totalling 7.1 km line of video whilst a total of 6.4 km line of video were collected over a depth range of 956–1889 m from the SE area. A total of 253 morphospecies were identified and catalogued.

#### Community analysis

**Fig 3:**
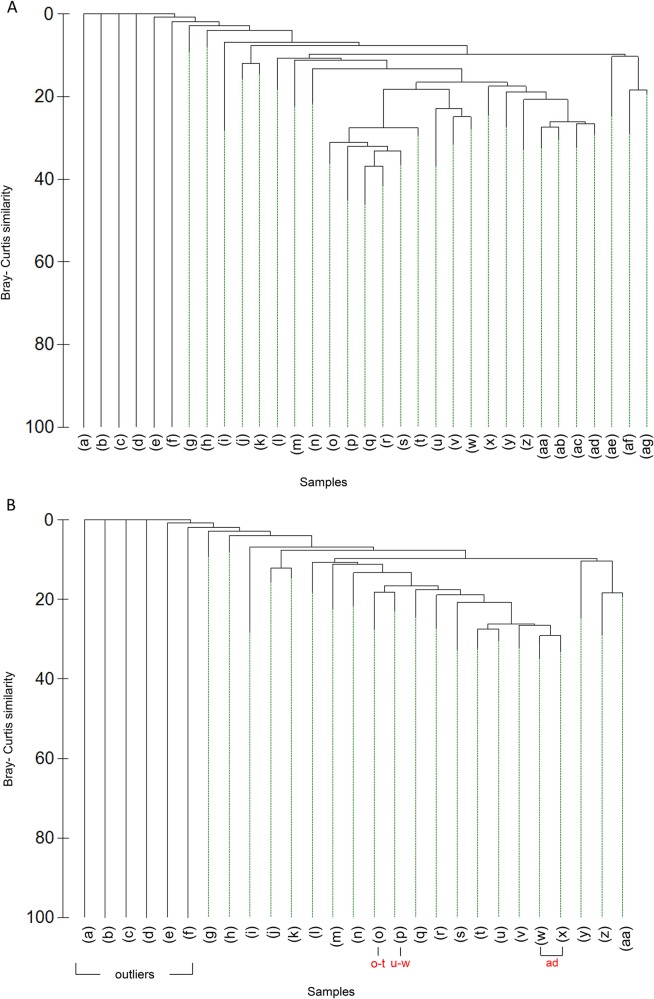
Dendrogram of hierarchical cluster analysis of species data, clusters identified using the SIMPROF routine (p < 0.01). Dendrogram (A) shows the SIMPROF cluster results prior to splitting and consolidation of clusters and (B) shows re-labelled clusters after splitting and consolidation. Those clusters that were combined or split from Fig. 3a are labelled in red. SIMPROF clusters are collapsed for ease of interpretation and are represented by green dashed lines. Grey lines represent SIMPROF identifying with no significant internal structure.

Thirty-three clusters (Fig 3A) were identified using the SIMPROF routine (p < 0.01), outlier clusters (a-f) and those which contained less than eight images (l, m, n, x, y, z, ab) were discarded. Upon examination of cluster “h” (which contained > 8 images) it was apparent that these images captured a microhabitat within the biotope of cluster “k” and was therefore not treated as a coherent cluster and were not investigated further. Cluster “ad” was subjectively divided on the basis of substratum (bedrock and dead coral framework) in line with current habitat classification schemes [See Fig 3A, cluster (ad)]. Six clusters [See Fig 3A, clusters labelled as (o-t)] were combined at a higher level of similarity, as were clusters u, v and w. Reasons for combining clusters o-t and u, v and w by moving up a node in the cluster analysis output are as follows: upon further examination of the underlying image data it was clear that the samples from the six clusters all occurred on the steep escarpment feature. The nature of the terrain affected how close the drop-frame camera could get to the seabed, thus making consistent sampling difficult. This was reflected in the samples, with varying sample sizes capturing fauna at different scales. These clusters were not deemed to be robust and were combined at a lower level of similarity. Given the problem with the changing size of field of view it could be argued that these data should be omitted. However, this would have resulted in no representation at all of the communities occurring on this type of terrain and seabed feature, which, given the aims of the study and the scarcity of data from seamounts, seemed the less favourable option. Thus, the data were retained but treated with a degree of caution. Clusters were re-labelled with the updated changes (Fig 3B) and these updated letters used throughout the remainder of the manuscript.

The SIMPER routine was repeated to incorporate the modified clusters as described above, and results, along with environmental data, can be found in S1 Table; full SIMPER results can be found in the S1 Appendix.

### Characterising mapping units (biotopes)

**Fig 4:**
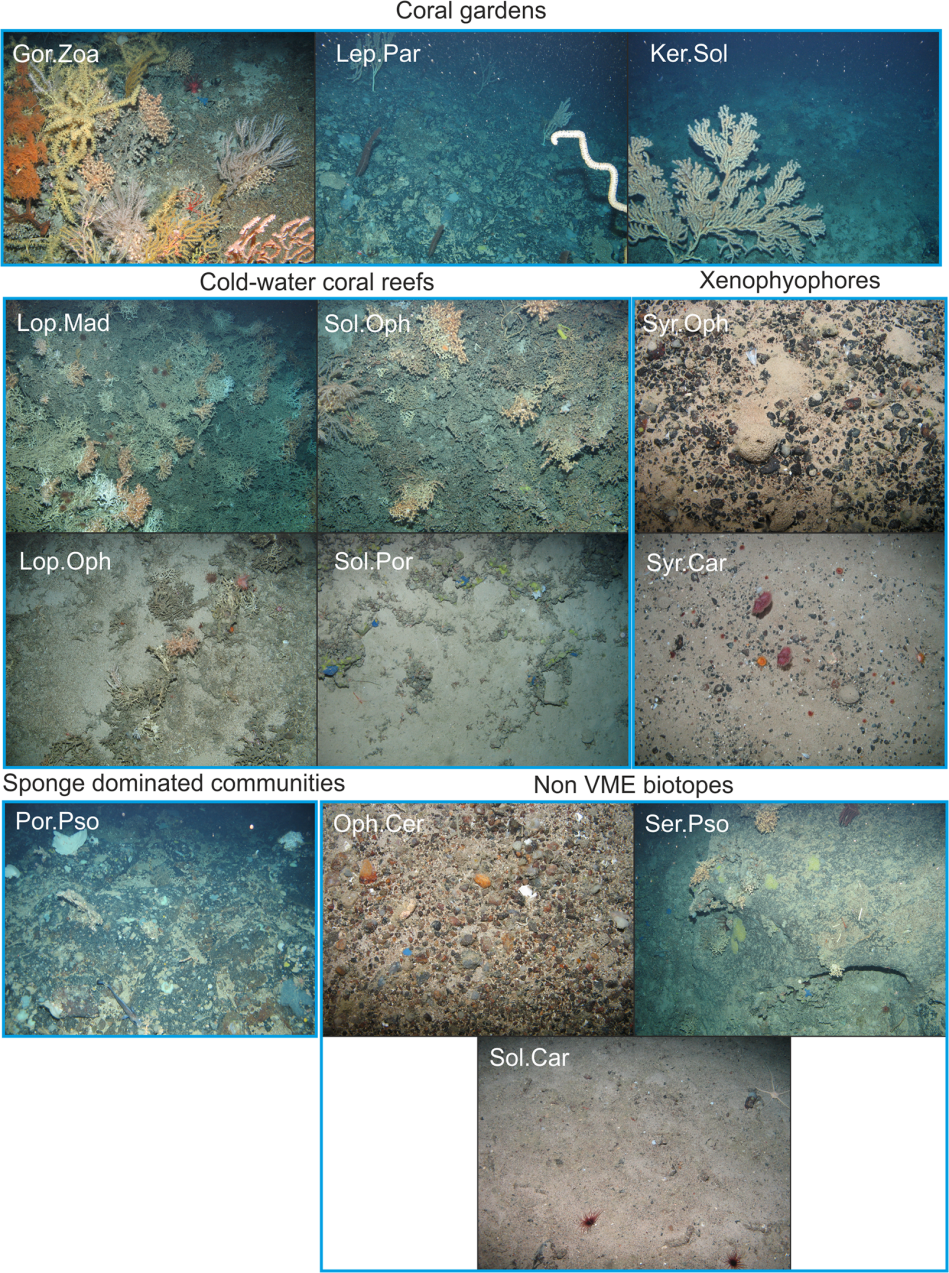
Example images of biotopes identified from multivarite cluster analysis. Refer to S1 Table for biotope details. g (Oph. Cer), i (Lop.Mad), j (Syr.Car), k (Syr.Oph), o (Por.Pso), p (Ser.Pso), t (Sol.Oph), u (Lop.Oph), w (Sol.Car), x (Sol.Por), y (Lep.Par), z (Ker.Sol), aa (Gor.Zoa). copyright JNCC, 2009.

In total 13 megabenthic assemblages were identified from the cluster analysis and related to available environmental data to describe distinct and useful mapping units [biotopes (see Fig 4, Table 2)]. Codes were allocated for those clusters that described biotopes; g (Oph. Cer), i (Lop.Mad), j (Syr.Car), k (Syr.Oph), o (Por.Pso), p (Ser.Pso), t (Sol.Oph), u (Lop.Oph), w (Sol.Car), x (Sol.Por), y (Lep.Par), z (Ker.Sol), aa (Gor.Zoa). The One-way ANOSIM test performed on the environmental data (depth and temperature) associated with each biotope revealed a significant difference in environmental conditions between biotopes (Global R = 0.652; p < 0.01). Forty three pairwise tests were significant.

**Table 2:** Summary of biotope data mapped from the videos.

Assemblage code	Cluster	No images analysed for defined biotope	Name of assemblage	Depth (m) & temperature (°C)	Associated geomorphic feature	Survey area	NW depth (m) & temperature (°C)	SE depth (m) & temperature (°C)
**Oph.Cer**	**g**	16	*Ophiomusium lymani* and cerianthid anemones on mixed substratum	1791–1889	Seamount flank	SE		1791–1889
				3.7–3.9				3.7–3.9
**Lop.Mad**	**i**	9	*Lophelia pertusa* reef	747–791	Cliff-top mounds	NW	747–791	
				8.83–9.02			8.83–9.02	
**Syr.Car**	**j**	15	Xenophyophores and caryophyllids on gravelly sand and mixed substratum	1714–1770	Seamount flank	NW	1714–1770	
				3.76–3.88			3.76–3.8	
**Syr.Oph**	**k**	22	Xenophyophores and ophiuroids on mixed substratum	1099–1544	Flank, radial ridge, escarpment	NW & SE	1076–1388	1099–1544
				4.37–7.9			5.1–7.9	4.3–7.26
**Por.Pso**	**o**	44	*Psolus*, caryophyllids and lamellate sponges on mixed, boulder and bedrock	854–1345	Escarpment	NW & SE	854–1345	994–1232
				5.1–9			5.1–9	6.2–8.2
**Ser.Pso**	**p**	26	Serpulids, encrusting sponges and *Psolus* on mixed substratum	813–1037	Summit, Escarpment	NW & SE	813–1037	956–1016
				8–9.1			8.18–9.05	8–8.32
**Sol.Oph**	**t**	30	*Solenosmilia variabilis* coral reef	1318–1351	Radial ridge	NW	1318–1351	
				5.16–5.42			5.16–5.42	
**Lop.Oph**	**u**	8	Predominantly dead, low-lying *L*.*pertusa* coral framework	758–994	Cliff edge, cliff-top mounds	NW & SE	758–814	972–994
				8.17–9.08			8.9–9.08	8.17–8.23
**Sol.Car**	**w**	21	*Solenosmilia variabilis* and encrusting sponges on bedrock	1270–1763	Parasitic cone, radial ridge,landslide/rockfall, escarpment	NW & SE	1270–1763	1496–1573
				3.77–5.8			3.76–5.88	4.33–4.76
**Sol.Por**	**x**	36	Predominantly dead, low-lying *Solenosmilia variabilis* coral framework with encrusting sponges	1267–1755	Radial ridge, parasitic cone, landslide/rockfall, flute	NW & SE	1266–1755	1496–1573
				3.76–5.88			3.76–5.88	4.33–4.76
**Lep.Par**	**y**	8	Coral garden with bamboo corals and antipatharians on bedrock	1724–1740	Parasitic cone	NW	1724–1740	
				3.87–3.89			3.87–3.89	
**Ker.Sol**	**z**	10	Coral garden with bamboo corals and *Solenosmilia variabilis* on bedrock	1542–1565	Radial ridge	SE	1542–1565	
				4.43–4.74			4.43–4.74	
**Gor.Zoa**	**aa**	24	Mixed corals and zoanthid coral garden	1311–1598	Radial ridge, parasitic cone	NW	1311–1598	
				4.23–5.67			4.23–5.67	

The table includes depth and temperature range of biotopes from the NW and SE study areas and associated geomorphic feature.

### Vulnerable Marine Ecosystems

#### Cold-water coral reefs

Four biotopes were defined that could be considered as cold-water coral reef, two are characteristic of summit reef and the other two of framework structures.

Live biogenic coral reef (Lop.Mad) was characterised by the reef building corals *Lophelia pertusa* (live colonies and framework) and *Madrepora oculata*, the pencil urchin *Cidaris cidaris*, anemones (Actiniaria sp.), decapods (Decapoda sp. 5) and the squat lobster *Munida sarsi*, gorgonian species and the antipatharian *Leiopathes* sp. (video footage). These findings broadly support those of previous studies.

Sol.Oph was characterised by framework and live *Solenosmilia variabilis*, ophiuroids (*Ophiactis* sp.) and a white encrusting sponge (Porifera encrusting sp. 42) on the *S*. *variabilis* framework. Non-sample images and video footage highlight the occurrence of a large ascidian species, a bamboo coral (Isididae sp. 2) associated with live growths of *S*. *variabilis* and a number of gorgonian species. The biotope is very similar to the *L*. *pertusa* reef (Lop.Mad), but is found deeper.

Two coral framework biotopes were identified on Anton Dohrn Seamount and varied in their composition and associated fauna. Biotope Lop.Oph was characterised by *L*. *pertusa* coral framework, *Madrepora oculata*, the pencil urchin *Cidaris cidaris*, ophiuroids (*Ophiactis*) and anemones (Actinaria sp., *Protanthea simplex*). Video footage revealed the large anemone *Phelliactis* sp. and the corkscrew antipatharian *Stichopathes* sp. to also be characterising species. While biotope Sol.Por was characterised by low-lying *S*. *variabilis* framework, a number of ophiuroid species (Ophiuroidea sp.2, Ophiuroidea sp.8, *Ophiactis*) and green encrusting sponges. Non-sample images and video suggested caryophyllids, blue encrusting sponges, the glass sponge *Aphrocallistes* sp. and the soft coral *Anthomastus grandiflorus* may also be abundant.

#### Xenophyophore aggregations

Two different xenophyophore assemblages were identified from Anton Dohrn Seamount. The biotope xenophyophores and ophiuroids on mixed substratum (Syr.Oph) was characterised by the xenophyophore *Syringammina fragilissima*, an unidentified ophiuroid species (Ophiuroidea sp. 1), a white encrusting sponge (Porifera encrusting sp.1), and Porifera massive globose sp. 12. This biotope occurred on mixed substratum (dominated by pebbles) on both sides of the seamount.

The biotope xenophyophores and caryophyllids on gravelly sand and mixed substratum (Syr.Car) was also characterised by xenophyophores (*Syringammina fragilissima*) but was distinguishable from the previous biotope by the presence of various anthozoan species. Other characterising species associated with this biotope were a solitary coral species (Cnidaria sp. 1) an unidentified ophiuroids species (Ophiuroidea sp.8) and *Ophiactis abyssicola*. Video observations suggested cerianthid anemones and pennatulids (*Pennatula phosphorea* and *Halipteris* sp.) may also be abundant throughout the biotope.

#### Coral gardens

The coral garden Lep.Par was characterised by the large bamboo corals *Lepidisis* sp. and the antipatharians *Parantipathes* sp., solitary cup corals (*Caryophyllia*), Porifera encrusting sp. 28 (white encrusting sponge) and *Psolus squamatus*.

The second coral garden biotope observed on Anton Dohrn Seamount (Gor.Zoa) was characterised by *Solenosmilia variabilis* framework, small growths of live *Solenosmilia variabilis* with an associated bamboo coral (Isididae sp. 2), ophiuroids (*Ophiactis*) Porifera encrusting sp. 6 and the soft coral *Anthomastus grandiflora*. A Zoanthidae species (sp.6) was found in abundance growing on gorgonian skeletons. Non-sample images and video observation suggest other conspicuous fauna to include the antipatharians *Antipathes* sp., *Leiopathes* sp., *Stichopathes* sp. and the glass sponge *Aphrocallistes* sp., and a number of gorgonian species

Ker.Sol was characterised by the large gorgonian *Keratoisis* sp. 2, small growths of *Solenosmilia variabilis*, cup corals *(Caryophyllia* sp. 2), Ophiuroidea sp. 4 and a blue encrusting sponge (Porifera encrusting sp. 6). Video footage revealed the presence of the false boarfish *Neocyttus helgae*, thus suggesting fast currents.

#### Sponge dominated communites

Biotope Por.Pso occurred on the steep escarpment which was comprised of bedrock outcrop with a boulder and cobble scree below. Characterising species as identified by SIMPER were the sessile holothurian *Psolus squamatus*, the ophiuroids *Ophiactis balli*, and encrusting sponges (Porifera encrusting sp. 22, Porifera encrusting sp. 28). Video observations also revealed this biotope to be characterised by lamellate sponges and the large conspicuous antipatharian coral *Leiopathes* sp.

#### Other ‘reef’ habitat under EU Habitats Directive

This biotope was characterised by small colonies of *S*. *variabilis*, a number of ophiuroid species (Ophiuroidea sp. 2, Ophiuroidea sp. 8), crinoids (Crinoidea sp. 1) *Caryophyllia* sp. 2 and encrusting sponges. The SIMPROF routine included this biotope with the dead framework and encrusting sponges biotope (Sol.Por), but was separated on the basis of substratum, in line with current habitat classification systems. Despite the same characterising species being present, it is important from a conservation perspective to know if it is a coral ‘reef’ or an area of bedrock with reef-like fauna. Video observation revealed the presence of the antipatharian *Bathypathes* and encrusting sponges.

#### Other habitats

Biotope Oph.Cer is characterised by the large ophiuroid *Ophiomusium lymani* and an unidentified species (unknown sp.29). Non-sample images and video revealed cerianthids to be a characterising species of this assemblage, as well as solitary corals (probably *Flabellum* sp.), stalked crinoids, holothurians and large anemones (Actinaria sp.16).

Biotope Ser.Pso was characterised by a number of sessile fauna including serpulid worms (Serpulidae sp. 1), encrusting sponges (Porifera encrusting sp. 1, Porifera encrusting sp. 28), the holothurian *Psolus squamatus*, globose sponges (Porifera massive globose sp. 12), ophiuroids (*Ophiactis abyssicol*, Ophiuroidea sp. 6) and Majidae sp. 1.

### Megabenthic diversity analysis

**Fig 5:**
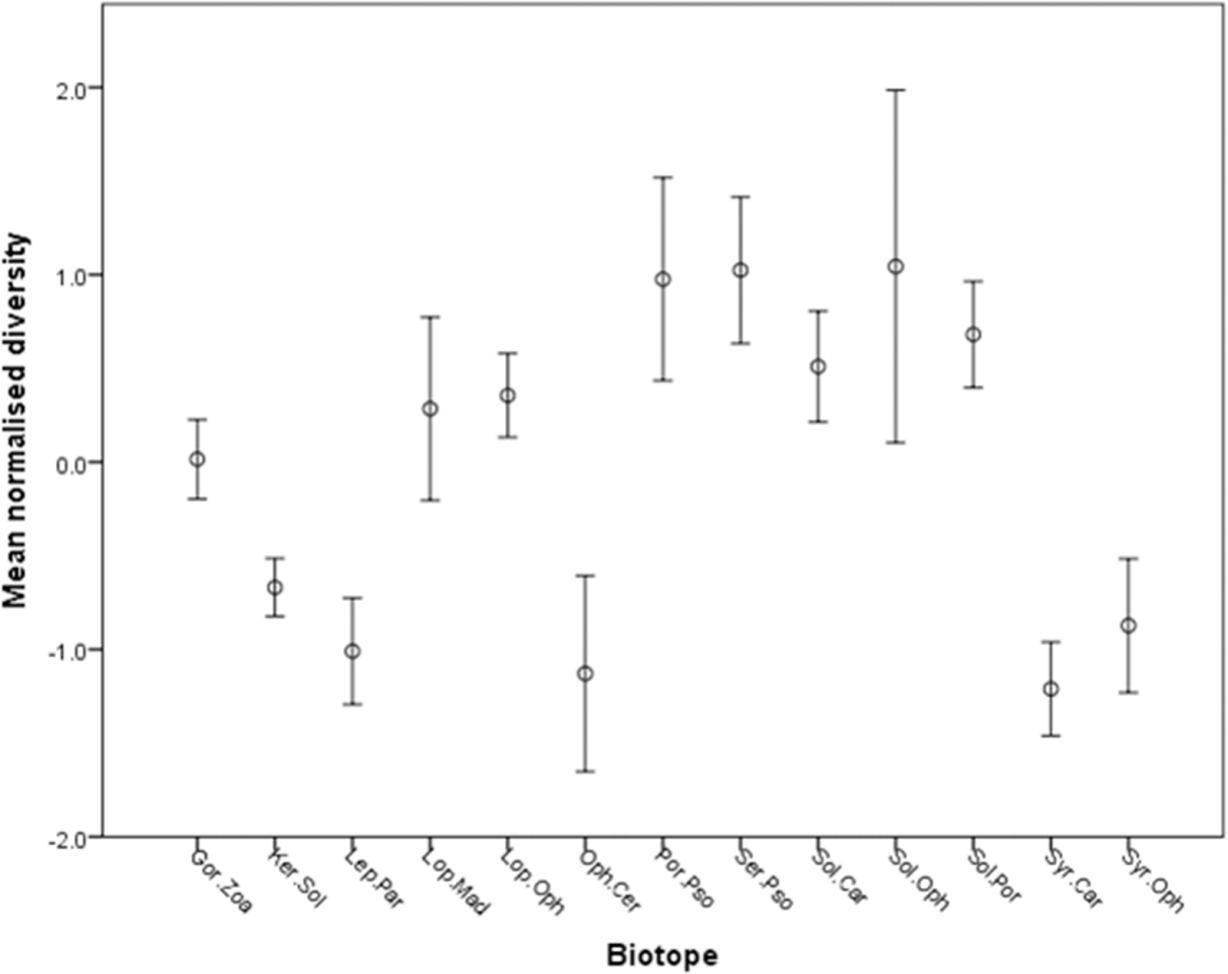
Mean normalised diversity and 95% confidence intervals of biotopes defined from Anton Dohrn Seamount.

Biotopes with the highest mean diversity are Por.Pso, Ser.Pso, and Sol.Oph. Those with the lowest mean diversity are Syr.Car, Syr.Oph, Oph.Cer, Lep.Par, Ker.Sol (Fig 5, Table 3). The One-way ANOSIM test revealed a significant difference in mean normalised diversity between biotopes (Global R = 0.564; p < 0.01). Forty-two pairwise tests were significant (p<0.05; Table 4). All soft sediment biotopes were found in the lower diversity group, while those biotopes associated with bedrock were in the higher diversity group (including the sponge dominated community Por.Pso). *Lophelia pertusa* reef were intermediate of these, while *Solenosmilia variabilis* reef are of comparable diversity to the bedrock biotopes. Two of the coral garden biotopes Lep.Par and Ker.Sol had lower diversity, comparable to the soft sediment biotopes, while the other coral garden Gor.Zoa had much higher diversity.

**Table 3:** Results of all diversity indices (mean) used in the multivariate ANOSIM.

Biotope	Species richness (Mean)	Simpson Reciprocal Index	Sobs	ICE (Mean)	Chao 2(Mean)	Jack 1 (Mean)	Jack 2 (Mean)	Bootstrap (Mean)
Gor.Zoa	12.5	2.4781	39.04	63.24	63.34	54.92	62.61	46.68
Ker.Sol	8.1	2.648619	15.71	44.97	46.86	35.81	39.98	30.71
Lep.Par	9.125	2.565343	15	34.82	38.47	30.25	31.66	26.9
Lop.Mad	15	3.678168	33.56	47.68	49.63	45.53	49.27	40.36
Lop.Oph	14	2.78942	48	51.76	54.9	50.21	54.08	44.34
Oph.Cer	5.176471	3.008829	21.36	38.45	40.03	29.84	33.92	25.09
Por.Pso	13.90909	2.627727	53.63	88.45	86.6	71.95	83.27	60.24
Ser.Pso	15.07407	3.973387	49.96	76.05	82.95	68.92	78.76	58.55
Sol.Car	17.09524	2.777351	43.2	66.14	68.2	59.97	67.71	51.7
Sol.Oph	15.7	1.893117	48.26	95.35	97.17	76.06	89.41	63.23
Sol.Por	17.81081	3.172699	48.13	72.21	79.39	65.79	75.37	56.32
Syr.Car	7.333333	2.516744	20.72	36.73	34.42	30.16	34.16	25.53
Syr.Oph	6	2.331484	27.39	56.44	56.36	35.41	42.34	28.79

**Table 4:** ANOSIM pairwise test results for multivariate diversity test.

	Gor.Zoa	Ker.Sol	Lep.Par	Lop.Mad	Lop.Oph	Oph.Cer	Por.Pso	Ser.Pso	Sol.Car	Sol.Oph	Sol.Por	Syr.Car
Ker.Sol	**0.809**											
	**(0.01)**											
Lep.Par	**0.854**	0.179										
	**(0.01)**	(0.48)										
Lop.Mad	0.308	0.298	0.458									
	(0.07)	(0.02)	(0.02)									
Lop.Oph	0.162	**0.609**	**0.751**	0.007								
	(3.06)	**(0.01)**	**(0.01)**	(3.38)								
Oph.Cer	**0.674**	0.276	-0.039	0.457	**0.650**							
	**(0.02)**	(0.05)	(7.45)	(0.01)	**(0.01)**							
Por.Pso	**0.653**	**0.850**	**0.912**	0.405	0.497	**0.828**						
	**(0.02)**	**(0.01)**	**(0.01)**	(0.13)	(0.07)	**(0.01)**						
Ser.Pso	**0.709**	**0.991**	**0.998**	**0.585**	**0.631**	**0.932**	0.186					
	**(0.02)**	**(0.01)**	**(0.01)**	**(0.01)**	**(0.02)**	**(0.01)**	(0.1)					
Sol.Car	0.315	**0.946**	**0.963**	0.378	0.243	**0.768**	0.393	0.395				
	(0.02)	**(0.01)**	**(0.01)**	(0.05)	(0.33)	**(0.02)**	(0.11)	(0.07)				
Sol.Oph	**0.650**	**0.739**	**0.710**	0.422	**0.599**	**0.667**	0.081	0.174	0.479			
	**(0.01)**	**(0.01)**	**(0.02)**	(0.07)	**(0.01)**	**(0.02)**	(0.91)	(0.33)	(0.03)			
Sol.Por	**0.782**	**0.999**	**1.000**	**0.571**	**0.647**	**0.935**	0.358	0.093	0.377	0.273		
	**(0.01)**	**(0.01)**	**(0.01)**	**(0.03)**	**(0.01)**	**(0.01)**	(0.03)	(1.07)	(0.05)	(0.03)		
Syr.Car	**0.904**	0.353	-0.063	0.554	**0.844**	-0.060	**0.925**	**0.998**	**0.983**	**0.724**	**1.000**	
	**(0.01)**	(0.06)	(8.7)	(0.02)	**(0.01)**	(9.43)	**(0.01)**	**(0.01)**	**(0.01)**	**(0.01)**	**(0.01)**	
Syr.Oph	**0.635**	-0.045	0.136	0.112	0.391	0.197	**0.798**	**0.969**	**0.832**	**0.722**	**0.985**	0.267
	**(0.04)**	(6.66)	(0.85)	(0.8)	(0.04)	(0.21)	**(0.02)**	**(0.02)**	**(0.01)**	**(0.01)**	**(0.01)**	(0.2)

Those in bold are significant (P<0.05).

### Distribution of biotopes in relation to meso-scale geomorphology

**Fig 6:**
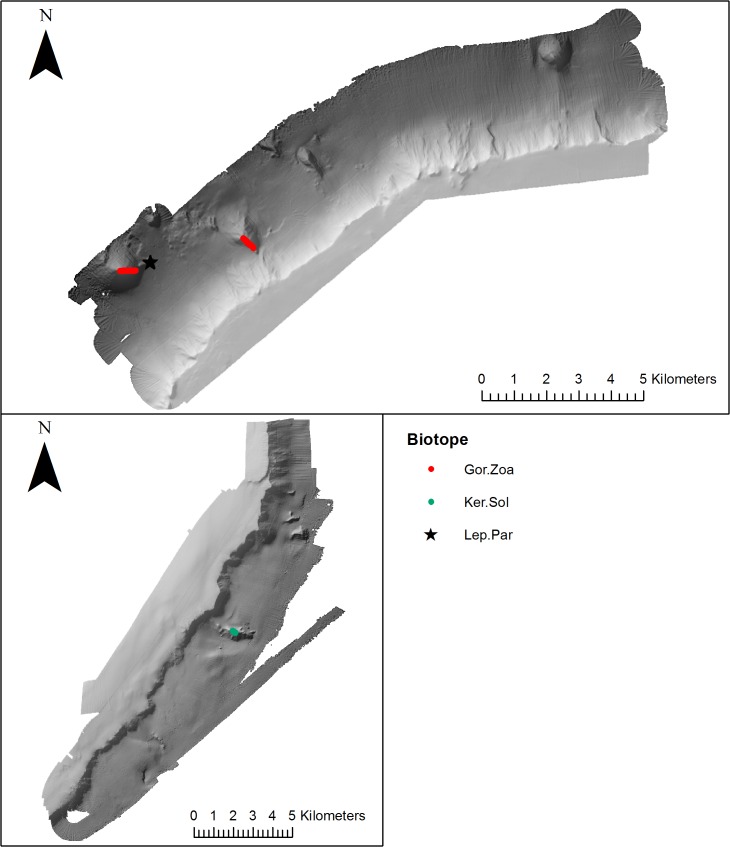
Distribution of coral garden biotopes (mapped using video footage) on the NW and SE side of Anton Dohrn Seamount. Refer to Fig 2 for bathymetric scale.

**Fig 7:**
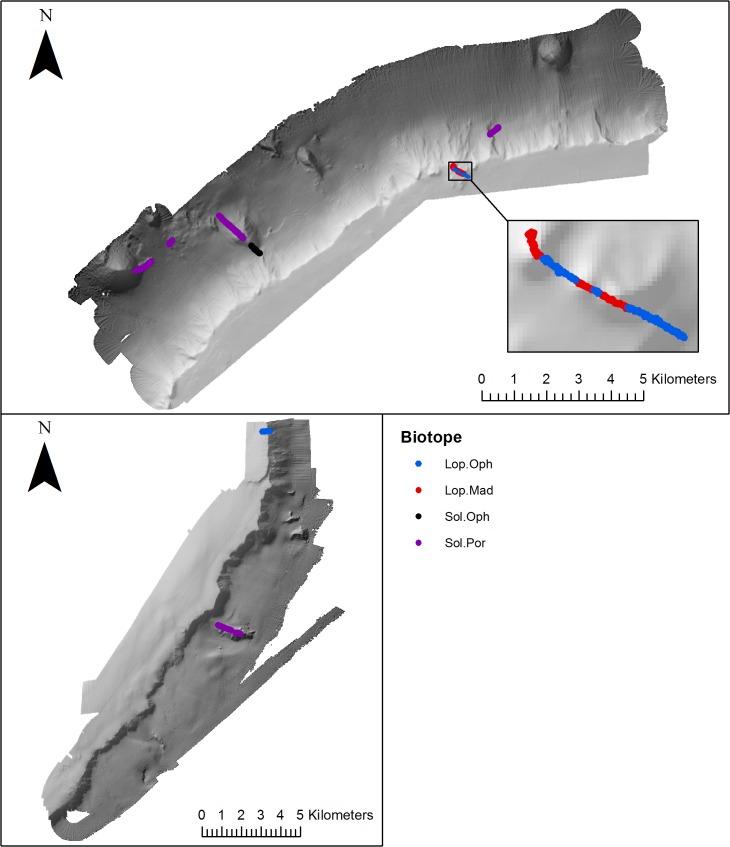
Distribution of cold-water coral reef (mapped using video footage) biotopes on the NW and SE side of Anton Dohrn Seamount. Refer to Fig 2 for bathymetric scale.

**Fig 8:**
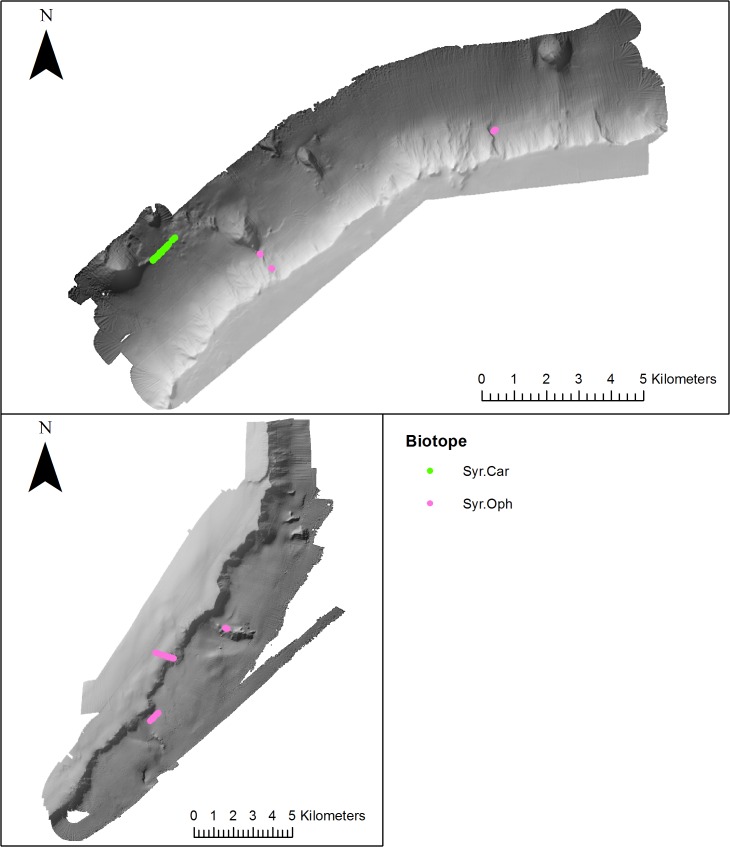
Distribution of xenophyophore communities (mapped using video footage) biotopes on the NW and SE side of Anton Dohrn Seamount. Refer to Fig 2 for bathymetric scale.

**Fig 9:**
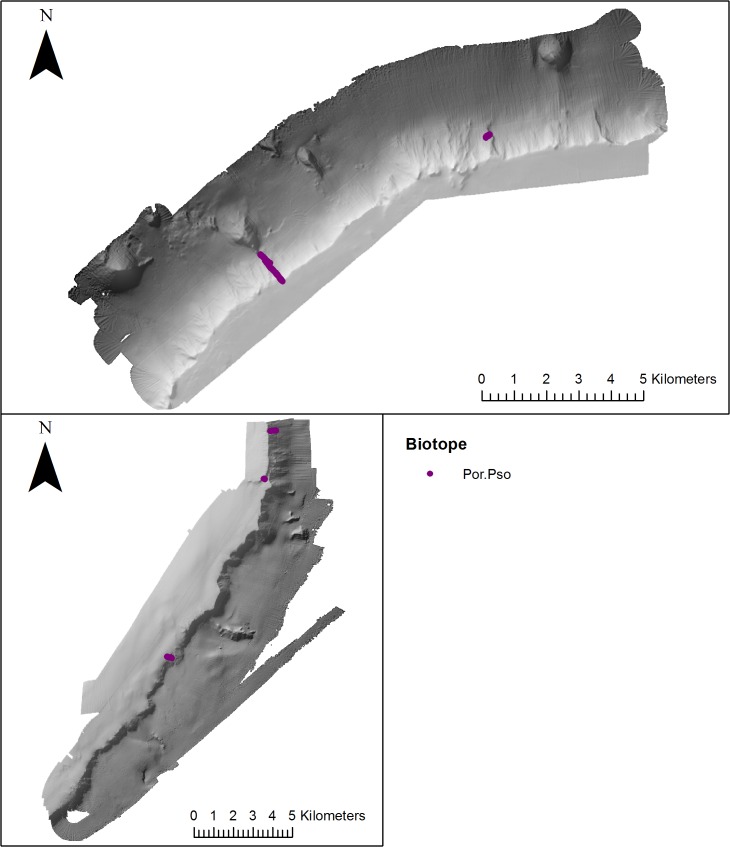
Distribution of sponge dominated communities (mapped using video footage) biotope on the NW and SE side of Anton Dohrn Seamount. Refer to Fig 2 for bathymetric scale.

**Fig 10:**
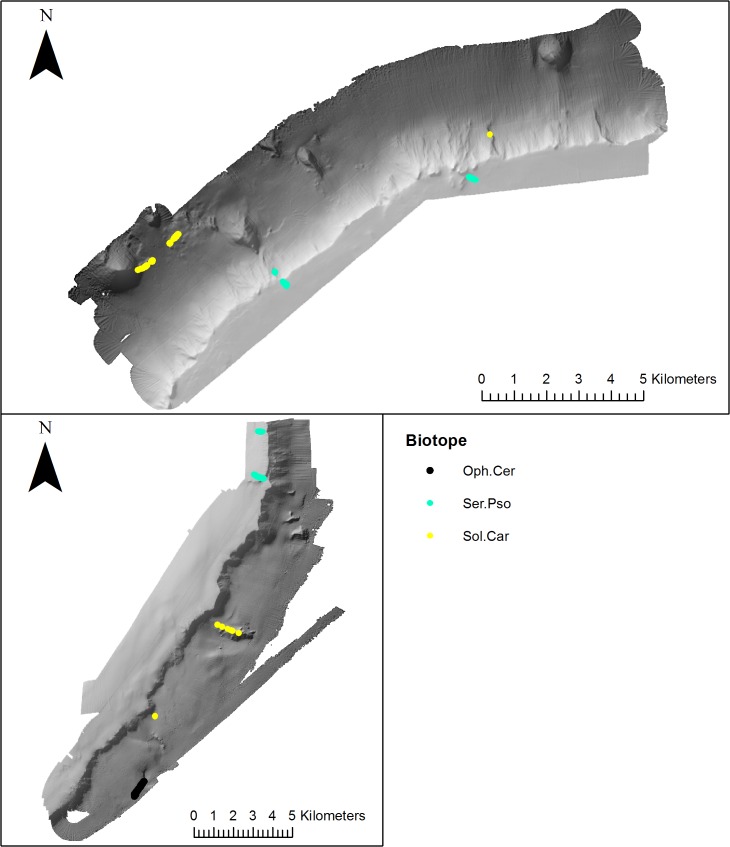
Distribution of non-VME biotopes (mapped using video footage) biotopes on the NW and SE side of Anton Dohrn Seamount. Refer to Fig 2 for bathymetric scale.

Qualitative assessment of biotope distribution, determined from visually classified video transect data (Table 2) revealed the occurrence of biotopes either side of Anton Dohrn Seamount. Thirteen biotopes were mapped from Anton Dohrn Seamount, eleven from the NW side and 8 from the SE (Fig 6–10). Of those thirteen, eleven fit the listed habitats definition and can be classed as habitats of conservation interest. Ten can be identified as VMEs (four coral reef, three coral gardens, two xenophyophore communities and one sponge dominated community), and one as bedrock reef under the EC Habitats Directive.

Three coral gardens were identified and mapped on distinct meso-scale geomorphological features (Fig 6). The coral garden characterised by the conspicuous gorgonian *Lepidisis* and the antipatharian *Parantipathes* sp.1 on bedrock (Lep.Par) occurred on a parasitic cone on the NW flank (1724–1740 m). The gorgonian dominated coral garden biotope Gor.Zoa only occurred on the NW side of the seamount on a parasitic cone and radial ridge feature (1311–1598 m), the third coral garden (Ker.Sol) characterised by the large conspicuous *Keratoisis* sp.2. bamboo coral and small growths of *Solenosmilia variabilis* was the only coral garden to occur on the SE side of the seamount, and was found on a radial ridge at a depth of 1542–1565 m.

*Lophelia pertusa* reef (Lop.Mad) was recorded on the summit of the cliff-top mounds on the NW side of the seamount at a depth of 747–791 m. Predominantly dead coral framework (Lop.Oph) occurred on the SE summit edge and between the cliff top mounds on the NW side, while *Solenosmilia* reef (Sol.Oph) was recorded at greater depths of 1318–1351 m on the radial ridge on the NW flank and dead, low-lying *S*. *variablis* reef on radial ridges (NW & SE), parasitic cone (NW), landslide/Rockfall (NW) and flute features on the NW flank (Fig 7).

The xenophyophore biotopes occurred on both the NW and SE side of the seamount (Fig 8), on the SE side of the summit edge (1104–1154 m) and on the flanks on both sides, although only on one distinct meso-scale feature; a radial ridge on the NW and SE side flank.

The sponge dominated community Por.Pso was observed on both the NW and SE side of the seamount (Fig 9) associated with the escarpment feature (854–1345 m). See Fig 10 for distribution of non-VME biotopes.

## Discussion

Seamounts are described as biodiversity hotspots and are listed as features of conservation interest, despite this there are few descriptions of biological assemblages from seamounts, particularly in the context of biotope mapping. Thirteen biotopes were identified from Anton Dohrn Seamount. Biotopes were considered against the specified restricted current definition of VMEs and OSPAR habitats [5, 38] and ten could be considered as VMEs and OSPAR habitats. Of these ten, four could be classified as cold-water coral reef, three as coral gardens, two as xenophyophore communities and one as a sponge dominated community. Although not assessed as VMEs using the restricted definition applied in this study the remaining three biotopes may still be considered VMEs when assessed against the list of criteria provided in the FAO guidelines [5]. Of these three, one could be considered as bedrock/stony reef under the EC Habitats directive.

### Descriptions of listed habitats for use as mapping units

#### Cold-water coral reefs

In this study the depth ranges of the two framework assemblages identified varied, with Lop.Oph biotope occurring much shallower (758–994 m) than Sol.Por (1267–1755 m), which may be explained by the known depth range of the dominant reef-building species. The latter assemblage appears to be subject to increased sedimentation, which is possibly the reason for a significantly lower proportion of live coral framework polyps observed than from the former assemblage. This may explain the abundance of encrusting sponge species on the *Solenosmilia* framework. Reef building scleractinia such as *Lophelia pertusa* are efficient at removing sediment from their polyps, and this ability can act as an indicator of the ‘health status’ of a reef [60]. If sediment is not removed, the accumulated sediment can smother the polyp and cause tissue loss. It is this exposure of bare skeleton which leave the coral vulnerable to fouling. It maybe that *Solenosmilia* is not as efficient as *Lophelia* at removing sediment, or that the hydrographic conditions vary between the localities, with slower currents depositing more sediment in the vicinity of the *Solenosmilia* reefs [61, 62].

Freiwald *et al*. [63] described the summit regions of cold-water coral mounds and live reef areas as supporting few permanently attached organisms, as the living corals are very successful in preventing fouling. Among those species that are permanently attached are the polychaete *Eunice norvegica*, the parasitic foraminiferan *Hyrrokkin sarcophagi*, and clusters of bivalves including *Delectopecten vitreus* and *Acesta excavata*. Howell *et al*. [33] describe an assemblage characterised by *L*. *pertusa*, *M*. *oculata*, hydroids, anemones, decapods, cerianthid anemones and echinoderms (ophiuroids and echinoids) from Hatton Bank, George Bligh Bank, Rockall Bank and the Wyville Thomson Ridge; and Davies *et al*. [34] describe a very similar biotope from the submarine canyons of the South Western Approaches (UK), characterised by live and dead *L*. *pertusa*, *M*. *oculata*, and Actiniaria sp.

Coral framework assemblages have been described by a number of authors: Wilson [64] describes an assemblage that was associated with dead coral debris as supporting a diverse range of fauna including bryozoans, anemones, calcareous polychaetes, bivalves, asteroids and echinoids, whilst Freiwald *et al*. [63] list gorgonians, actinians and sponges as conspicuous and abundant megafauna within this habitat, whereas on a smaller scale hydrozoans, bivalves, brachiopods, bryozoans and barnacles are prevalent. Howell *et al*. [33] describe an assemblage characterised by *L*. *pertusa* framework, halcampoid anemones, encrusting bryozoans, encrusting sponges, squat lobsters, serpulid polychaetes, echinoderms (ophiuroids and asteroids), cup corals and ascidians. Coral framework is known to be more diverse than the living part of the reef [65] and it has been suggested that the reason for this may be that live coral tissue prevents sessile epibiotic species from attaching to the framework [66]. The hard coral skeleton provides a surface for attachment of associated fauna [29].

#### Xenophyophore aggregations

Large epifaunal xenophyophores increase habitat heterogeneity of deep-sea sediments and could serve the role of a structural habitat in providing: hard substratum for epifaunal species, refuge from predators, microhabitats for mating, reproduction and nursery functions, elevated positions for suspension feeders, and increased food availability to deposit feeders resulting from the deposition of fine particles [67, 68]. The xenophyophores increase local biodiversity and represent a unique habitat on deep-sea soft sediments as many of the associated species do not occur on the surrounding seafloor where xenophyophores are absent [29].

Whilst many authors have described the distribution of xenophyophore aggregations [69, 70] there have been few descriptions of them in terms of an assemblage. The two xenophyophore assemblages identified on Anton Dohrn Seamount were found on gravelly sand or mixed substratum on the deep flanks of the seamount associated with, or proximal to, positive topographic features such as flutes on the cliff edge or cliff top mounds. These assemblages are similar to those described by Narayanaswamy *et al*. [71] who identified xenophyophores and ophiuroids as being the dominant fauna between 980–1004 m on the Hebrides continental slope and between 798–835 m on Hatton Bank; and xenophyophores, sea pens and solitary corals (probably *Flabellum* sp.) between 1739–1963 m on the NW flank of Anton Dohrn Seamount and xenophyophores, cerianthids and caryophyllids on George Bligh Bank (1112–1154 m) [72].

#### Coral gardens

Coral gardens are listed under the OSPAR Agreement as ‘threatened and/or declining species and habitats’ [3] and are defined as ‘a habitat which has a relatively dense aggregation of individuals or colonies of one or more coral species which can occur on a wide range of soft and hard substrates’ [73]. In the context of hard substratum this habitat has been described as being dominated by gorgonian, stylasterid and/or antipatharian corals [74] and can develop on exposed bedrock, boulders or cobbles [75]. Current coral garden definitions note, “the definition does not encompass deeper-water habitats where sponges (deep-sea sponge aggregations) dominate”, but continue to list deep-sea sponge species being associated with coral garden habitats.

Coral garden with bamboo corals and antipatharians on bedrock. Wienberg *et al*. [76] describe a diverse ‘discrete live coral colonies’ assemblage from the Franken Mound on western Rockall Bank associated with ridge features on the eastern and western flanks at a depth of 650–675 m. The assemblage is dominated by gorgonians (*Acanthogorgia armata*), antipatharian corals (including *Bathypathes* sp., *Stichopathes* cf. *gravieri*, *Leiopathes* sp. and *Parantipathes* sp.), a number of soft coral species (including *Anthomastus* and *Capnella glomerata*), stylasterid corals and associated megafauna. They noted that scleractinian corals were sparse with only *L*. *pertusa* observed. Another obvious difference is the presence/absence of stylasterid corals and the relative abundance of *L*. *pertusa* which may be due to *L*. *pertusa* out-competing the stylasterid corals [77]. It is possible that the biotope observed on Anton Dohrn Seamount is a deeper version (1724–1740 m) of the assemblage described by Wienberg *et al*. [76].

Mixed corals and zoanthid coral garden. Coral gardens appear to provide a suitable habitat for a diverse range of fish including the false boarfish *Neocyttus helgae*, *Lepidion eques*, and orange roughy *Hoplostethus atlanticus* all of which were observed on video in this biotope, however no quantitative analysis has been undertaken to assess any statistical relationships between fish and habitat; but interestingly orange roughy were only observed associated with this coral garden biotope, despite transects undertaken elsewhere on the seamount at comparable depths. The bathypelagic false boarfish is a good indicator species for coral habitats as they have a facultative relationship with fan and whip octocoral-dominated habitats [78]. Their occurrence is thought to be indicative of a strong current regime [79].

Coral garden with bamboo corals and *Solenosmilia variabilis* on bedrock. Little has been documented regarding the distribution of coral garden habitats; many studies have identified the distribution of coral species which have the potential to form coral gardens, e.g. Bruntse and Tendal [80] described the distribution of gorgonians around the Faroe Islands and Grasshoff [81–87] reported the distribution of gorgonians, antipatharians and pennatulids in the NE Atlantic; although few authors have described coral garden assemblages. One of the most diverse coral garden habitats reported to date is from the Aleutian Islands and is dominated by gorgonians and stylasterid corals [88]. A review by OSPAR [73] summarised the occurrence/potential of coral garden habitats in the NE Atlantic. These include seamounts in the Azores which were dominated by large gorgonians and antipatharian corals, Le Danois Bank (Spain) which was characterised by the large gorgonian *Callogorgia verticillata* [35, 89], and the Mid-Atlantic Ridge [90]. Buhl-Mortensen *et al*. [29] refer to shallow (200 m) coral gardens characterised by *Paragorgia arborea* and *Primnoa resedaeformis* offshore Norway. Durán Muñoz *et al*. [91] identified areas of bedrock outcrop on the western flank of Hatton Bank from multibeam echosounder data, and results from dredge samples suggested that these outcrops provide suitable substratum for cold-water corals which may be potential coral gardens. Additional longline survey data identified a number of species associated with these areas as *L*. *pertusa*, *Madrepora oculata*, seafans, bamboo corals (*Acanella* sp.), antipatharians, stylasterids corals and glass sponges.

The coral gardens found on Anton Dohrn Seamount are the first to be described from UK waters. It appears that the coral framework in the Gor.Zoa biotope is acting as a substratum for the colonisation of other coral species, while Lep.Par and Ker.Sol are using bedrock as a point of attachment. It may be that the hydrodynamic conditions and substratum availability influence the distribution of these three biotopes.

#### Sponge dominated community

Por.Pso has not been described previously from the deep sea but is similar to that observed along a bedrock escarpment on Rockall Bank (~350–600 m) (Howell, unpublished) and appears to be a deeper version occurring along the steep break of the slope. The assemblage on Rockall Bank is characterised by large lobose sponges, stylasterid corals, encrusting sponges and the pencil urchin *Cidaris cidaris*, whilst the newly described assemblage from Anton Dohrn Seamount is characterised by lamellate sponges, large conspicuous coral (antipatharians), caryophyllids, small growth of *L*. *pertusa* and encrusting sponges. The main difference between these assemblages is the absence of stylasterid corals associated with the assemblage from Anton Dohrn Seamount, and may be because they are being out-competed by scleractinians that are more able to adapt to variable conditions [77]. While this biotope is characterised by a number of coral species, they are not the dominant species; lamellate and encrusting sponges are more abundant than corals. The current OSPAR definition for coral gardens does not include those habitats where sponges dominant [73], thus this biotope cannot be classed as a coral garden.

#### Other ‘reef’ habitat under EU Habitats Directive

One biotope was identified as potential bedrock ‘reef’ habitat under the EU Habitats Directive.

The Solenosmilia variabilis and encrusting sponges on bedrock biotope is similar to that described by Howell *et al*. [33] as ‘discrete coral (*Lophelia pertusa*) colonies on hard substratum’ from the Wyville Thomson Ridge and Hatton Bank at an average depth of 637 m. This assemblage differs to that described by Wienberg *et al*. [76] in terms of the relative proportion of corals species; with a lower abundance of conspicuous gorgonian and antipatharians species which are replaced by small growths of *S*. *variabilis*. Under the criteria set out by OSPAR, this biotope was not considered a coral garden due to the lower abundance of coral species.

#### Other habitats

*Ophiomusium lymani* and cerianthid anemones on mixed substratum. The long armed ophiuroid *Ophiomusium lymani* is known to occur in deep water and has been previously described by a number of authors (e.g. [92, 93]) as occurring in association with the bamboo coral *Acanella arbuscula* in the lower bathyal depths (1920–2500 m) in the Rockall Trough. Narayanaswamy *et al*. [71] describe an *Ophiomusium* assemblage at 1420 m on the NW flank of Anton Dohrn Seamount associated with echinoids (probably *Echinus affinis*), solitary corals and the soft coral *Anthomastus grandiflorus*, and an *Ophiomusium* and *Echinus affinis* assemblage at 2025–2180 m on the Hebrides continental slope, associated with solitary polyps (possibly *Flabellum* sp.). A deep *O*. *lymani* assemblage was observed from the moat of Rosemary Bank associated with unidentified annelid species, echinoids (*Echinus* sp.), *Psolus squamatus* and brachiopods on mixed cobble and pebble substrate (J. Davies, unpublished).This newly described biotope differs from previously described assemblages and was characterised by cerianthids, stalked crinoids, solitary corals (probably *Flabellum* sp.), large anemones and holothurians.

Serpulids, encrusting sponges and *Psolus* on mixed substratum. A similar assemblage has been described from Rockall Bank by Wienberg *et al*. [76]. They describe a dropstone associated community characterised by serpulid worms, bryozoans and *Psolus* sp. Howell *et al*. [33] also describe a similar assemblage associated with mixed substrate (pebbles-boulders) and bedrock characterised by saddle oysters, *Psolus squamatus*, white encrusting sponges, serpulid worms and *Munida* sp.

### Diversity of biotopes

The overall diversity captured by undertaking a multivariate diversity ANOSIM test suggests there are differences in diversity between the biotopes. The xenophyophore biotope Syr.Car had the lowest mean diversity of all thirteen biotopes. The documented enhanced diversity of organisms associated with xenophyophore aggregations is found within the metazoan macrofaunal compartment, which was not captured by the video and image based methods used in the present study. Sediments adjacent to large xenophyophore tests contain significantly more metazoan macrofauna than surrounding sediments [67, 94]. Incidental observations suggest that the tests of xenophyphores and large agglutinated foraminifera also provide microhabitats for small meiofaunal-sized metazoans [95] and foraminifera [96–98].

Of the three coral gardens biotopes the highest mean diversity was observed for the Gor.Zoa (Mixed corals and zoanthid coral garden) biotope that was also the shallowest occurring of the three at 1311-1598m. This biotope was observed on *S*. *variabilis* coral framework whereas both Ker.Sol and Lep.Par were observed on bedrock. The presence of the coral framework may provide structural complexity resulting in increased diversity. Interestingly, two of the coral garden biotopes Lep.Par and Ker.Sol (on bedrock) had comparable diversities to the soft sediment biotopes (Syr.Car, Syr.Oph, Oph.Cer), which had the lowest epifaunal diversities. This observation is likely to be an artefact as a result of variation in the size of the field of view of images taken on different terrains. On flat terrain, i.e. soft sediment, it is easy to land the camera, while of steeper terrain such as that of some of the bedrock areas samples, it is not possible to land the camera—thus the estimates of diversity must be treated with some caution for those areas where the camera could not be landed and thus had a variable size of the field of view.

The *S*. *variabilis* dominated cold-water coral reef biotopes [Sol.Oph (live reef summit) and Sol.Por (framework slopes)] although similar were generally of higher diversity than the functionally equivalent *L*. *pertusa* dominated cold-water coral reef biotopes [Lop.Mad (live reef summit) and Lop.Oph (framework slopes)]. The *Lophelia* dominated reef was generally found much shallower than the *Solenosmilia* dominated reef [747–791 m vs. 1318–1351 m live summit reef; 758–994 vs. 1267–1755 m framework slopes]. Very little data are available on the faunal composition of *S*. *variabilis* reefs. However, the fact that these reefs occur deeper than *L*. *pertusa* reefs in the study area may in part explain the increased diversity observed. In the North Atlantic parabolic patterns of species diversity with depth have been demonstrated for a variety of macrofaunal and megafaunal taxa, with maximum diversity occurring at mid slope depths (1800–2300 m) [99–105]. As *S*. *variabilis* reefs are closer to the depth of the general diversity peak it might be expected that they would show higher diversity than their shallower equivalents. However, this relationship does not hold across the other biotopes described. For the xenophyophore biotopes the shallower Syr.Oph supported higher diversity than Syr.Car. Of the three coral gardens biotopes again the shallower occurring Gor.Zoa supported the highest diversity. Of the two bedrock reef biotopes the shallower occurring Por.Pso (854–1345 m) was more diverse than Sol.Car which occurred deeper (1270–1763 m). The observed higher diversity for *S*. *variabilis* reef may simply be a result of a greater percentage of coral framework observed on these reefs than the *L*. *pertusa* reefs. The framework zones of reefs are more diverse than the live summits [65, 106].

### Relationship between ‘biotopes of conservation interest and meso-scale geomorphological features

#### Cold-water coral reef

In this study *L*. *pertusa* reef was associated with cliff top mounds and *S*. *variabilis* with radial ridges on the NW side of Anton Dohrn Seamount over a depth range of 747–791 and 1318–1351 m respectively. The findings of the *L*. *pertusa* reef support those of earlier studies which found that the largest reefs occur in depths between 500–1200 m [107, 108] and may be associated with topographic features such as ridges (Sula Ridge), escarpments (Pelagia Mounds) and channels (Hovland Mounds) [108–110]. Few data are available on geomorphological associations of *S*. *variabilis* reef but they are likely to be similar to *L*. *pertusa*. These relationships most likely reflect both the substratum and hydrodynamic requirements of reef habitat development [37]. Reef habitat forms in areas of enhanced turbidity, within a narrow density envelope, with high current velocities that prevent local sedimentation but provide enhanced encounter rates with food particles [111–113]. These conditions must be stable over long periods of time to allow reef development [111]. Factors driving the spatial distribution of reefs have been related to various oceanographic processes, including internal tides [107], rectification of diurnal tides and Taylor column formation [114], rapid down-welling of surface water caused by hydraulic control of tidal flow and advection of deep bottom water [115], again all thought to result in enhanced delivery of suspended particles to the corals [116]. While there are no data on the fine-scale oceanography of the Anton Dohrn Seamount, these types of oceanographic processes are likely to occur in areas of topographic complexity such as around mounds and on radial ridges. On a broader scale the Anton Dohrn Seamount is thought to support the development of Taylor Columns [117] known to be important in determining reef distribution [115].

#### Xenophyophore communities

The xenophyophore assemblages observed on Anton Dohrn Seamount were either associated with geomorphic features [flank, cliff edge (edge of seamount summit) and radial ridges)] or were in close proximity (< 100 m) to geomorphic features (between cliff edge and flute feature, between parasitic cone and landslide). Previous studies have shown they are often found in areas with enhanced organic carbon fluxes, such as beneath highly productive surface waters, on sloped topography, or near certain topographic features such as caldera walls, basalt outcrops, or on the sides of sediment mounds and small ridges [70, 94, 118, 119]. Rogers [8] suggested that this may be a result of topographically-enhanced currents or high concentrations of suspended matter associated with these regions, which provide an increased food supply for suspension feeding organisms such as xenophyophores.

#### Coral gardens

The OSPAR definition of coral gardens is very broad, and the habitat in terms of biodiversity and densities of associated species can vary with region, hydrography, topography, substratum and depth [73]. For these reasons OSPAR states that a more precise description within regional seas is needed as is the need to establish relationships with features, substratum and depth which can be used as proxies for identifying and mapping these vulnerable habitats.

The coral gardens observed on Anton Dohrn Seamount occur on distinct topographical features along the crest of the parasitic cones and radial ridges on the NW and SE flank. The coral garden with bamboo corals and antipatharians on bedrock (Lep.Par) occurred on the crest of a parasitic cone on the NW flank, the coral garden with bamboo corals and *Solenosmilia variabilis* (Ker.Sol) on bedrock occurred on a radial ridge on the SE flank, and the Mixed corals and zoanthid coral garden (Gor.Zoa) on both the radial ridge and parasitic cone on the NW flank.

The occurrence of these assemblages is most likely a result of the presence of favourable hydrodynamic conditions and suitable substratum in these areas. Gorgonians settle on hard substrata, and availability of hard substratum can be a limiting factor to their distribution [120]. Water motion is also one of the primary factors influencing the distribution of gorgonians [120, 121] because of its role in delivering food [122], removing CO_2_ [123] and preventing sedimentation. The elevated position provided by raised topographical features, in this case parasitic cones and radial ridges, provide optimal conditions for gorgonian settlement and growth.

#### Sponge dominated community

The sponge dominated community occurred on the steep escarpment encircling the seamount. The occurrence of these assemblages is most likely a result of the presence of favourable hydrodynamic conditions and suitable substratum in these areas.

## Conclusions

Anton Dohrn Seamount hosts a diverse range of biotopes, some of which have been described from other megahabitat features, such as banks and submarine canyons. Sampling of distinct geomorphological features identified eleven biotopes that fit with current definitions of those of conservation concern under FAO, OSPAR and EC Habitats Directive; ten of these are identified as VMEs.

This work not only provides much needed descriptions of deep-sea biotopes, which is important for the identification and subsequent protection of VMEs, but also provides new insights into their potential associations with meso-scale geomorphic features which may be used to map these habitats across broad areas. Listed habitats such as coral gardens, *Lophelia pertusa* reef and bedrock reef habitats were found on distinct topographical features including cliff-top mounds, parasitic cones and radial ridges; and xenophyophore assemblages were found either on geomorphic features or in close proximity (<100 m). There are therefore indications that some biotopes of conservation concern may show some relationship to meso-scale geomorphological features however, further work is needed to test this relationship. This work highlights the need for more comprehensive definitions of listed habitats such as coral gardens and VMEs to aid in identification and ultimately protection of these habitats.

## Supporting Information

S1 Appendix Multivariate SIMPER results. SIMPER results for biotopes defined using multivariate cluster analysis, species in bold are characterising species.
(DOCX)

S1 Table Results of multivariate cluster analysis. Clusters identified using the SIMPROF routine, SIMPER similarity, environmental variables and characterising species for each cluster identified.
(DOCX)
