# Supplementary material for: Benthic Assemblages of the Anton Dohrn Seamount (NE Atlantic): Defining Deep-Sea Biotopes to Support Habitat Mapping and Management Efforts with a Focus on Vulnerable Marine Ecosystems
Source: PLoS One. 2015 May 18;10(5):e0124815. doi: 10.1371/journal.pone.0124815 (PMC4436255; doi:10.1371/journal.pone.0124815)
Supplement: S1 Appendix — SIMPER results for biotopes defined using multivariate cluster analysis, species in bold are characterising species. (DOCX) [file pone.0124815.s001.docx]

**S1 Appendix. Multivariate SIMPER results.** SIMPER results for biotopes defined using multivariate cluster analysis. Characterising species are in bold.

***Group a***

Less than 2 samples in group

***Group b***

Less than 2 samples in group

***Group c***

Less than 2 samples in group

***Group d***

Less than 2 samples in group

***Group e***

Less than 2 samples in group

***Group f***

Less than 2 samples in group

***Group g (Oph.Cer)***

Average similarity: 21.35

Species Av.Abund Av.Sim Sim/SD Contrib% Cum.%

***Ophiomusium lymani* 0.06 11.53 0.97 53.98 53.98**

**Unknown sp. 29 0.04 3.84 0.40 18.01 71.98**

**Crinoidea sp. 7 0.03 2.78 0.38 13.02 85.00**

Halcampoididae sp. 3 0.01 0.64 0.21 3.00 88.00

Ophiuroidea sp. 2 0.01 0.45 0.20 2.09 90.09

*Caryophyllia* sp. 3 0.01 0.41 0.21 1.91 92.00

*Pandalus borealis* 0.01 0.34 0.21 1.59 93.59

*Syringammina fragillissima* 0.01 0.25 0.15 1.18 94.77

Ophiuroidea sp. 8 0.02 0.24 0.15 1.11 95.87

*Caryophyllia* sp. 2 0.01 0.16 0.15 0.77 96.64

Porifera encrusting sp. 28 0.01 0.12 0.15 0.57 97.21

Holothuroidea sp. 4 0.01 0.12 0.09 0.56 97.77

Porifera encrusting sp. 40 0.00 0.10 0.15 0.46 98.23

Actiniaria sp. 16 0.00 0.09 0.09 0.40 98.63

Ophiuroidea sp. 1 0.01 0.08 0.09 0.37 99.01

Porifera boring sp. 1 0.00 0.07 0.09 0.33 99.33

*Echinus acutus* 0.01 0.06 0.09 0.26 99.59

Crinoidea sp. 1 0.01 0.05 0.09 0.22 99.81

Porifera encrusting sp. 1 0.00 0.04 0.09 0.19 100.00

***Group h***

Average similarity: 14.51

Species Av.Abund Av.Sim Sim/SD Contrib% Cum.%

**Porifera encrusting sp. 1 0.02 7.55 0.99 52.07 52.07**

**Serpulidae sp. 1 0.03 4.47 0.36 30.83 82.90**

**Actiniaria sp. 9 0.02 1.23 0.21 8.45 91.34**

Porifera encrusting sp. 3 0.00 0.20 0.18 1.39 92.73

Porifera branching-erect sp. 2 0.01 0.20 0.09 1.37 94.11

Porifera encrusting sp. 31 0.00 0.20 0.09 1.35 95.46

*Caryophyllia* sp. 1 0.01 0.18 0.09 1.24 96.69

Anomiidae sp. 1 0.01 0.12 0.09 0.81 97.50

*Psolus squamatus* 0.01 0.10 0.09 0.67 98.17

Porifera encrusting sp. 25 0.01 0.09 0.12 0.60 98.77

Cerianthidae sp. 1 0.00 0.08 0.09 0.54 99.31

Porifera encrusting sp. 22 0.00 0.05 0.09 0.37 99.69

Porifera encrusting sp. 28 0.00 0.03 0.09 0.20 99.89

*Lophelia pertusa* 0.00 0.02 0.09 0.11 100.00

***Group i (Lop.Mad)***

Average similarity: 35.22

Species Av.Abund Av.Sim Sim/SD Contrib% Cum.%

***Madrepora oculata* 0.23 8.76 1.09 24.88 24.88**

***Lophelia pertusa* (dead structure) 0.15 4.51 1.11 12.82 37.70**

**Decapoda sp. 5 0.08 4.42 1.43 12.54 50.24**

***Cidaris cidaris* 0.07 3.64 1.70 10.34 60.59**

***Lophelia pertusa* 0.09 2.96 0.88 8.40 68.99**

**Actiniaria sp. 0.06 2.73 1.35 7.76 76.75**

*Acanthogorgia armarta* 0.04 1.53 0.71 4.33 81.08

*Munida sarsi* 0.04 1.29 0.70 3.66 84.75

*Protanthea simple* 0.05 1.05 0.37 2.99 87.74

Porifera encrusting sp. 15 0.10 0.94 0.27 2.66 90.40

*Leiopathes* sp. 1 0.03 0.66 0.45 1.88 92.29

Actiniaria sp. 9 0.03 0.59 0.43 1.66 93.95

Corallimorphidae sp. 2 0.03 0.35 0.23 0.98 94.93

Gastropoda sp. 1 0.02 0.33 0.37 0.93 95.86

Ascidiacea sp. 2 0.01 0.24 0.28 0.67 96.53

*Caryophyllia* sp. 2 0.01 0.21 0.29 0.61 97.13

Cerianthidae sp. 1 0.02 0.21 0.28 0.60 97.73

*Ophiactis balli* 0.02 0.13 0.19 0.36 98.09

Serpulidae sp. 1 0.01 0.11 0.19 0.31 98.40

*Stichastrella rosea* 0.01 0.10 0.20 0.30 98.70

*Henricia sanguinolenta* 0.01 0.09 0.20 0.25 98.95

*Margarites* sp. 1 0.01 0.08 0.20 0.24 99.18

Halcampoididae sp. 3 0.01 0.05 0.11 0.14 99.33

Ophiuroidea sp. 2 0.01 0.05 0.11 0.13 99.46

Hydrozoa (bushy) 0.02 0.04 0.11 0.12 99.58

*Ophiactis abyssicola* 0.01 0.03 0.11 0.09 99.67

*cf. Antipathella* sp. 1 0.01 0.03 0.11 0.09 99.76

*Psolus squamatus* 0.01 0.03 0.11 0.09 99.85

*Chaceon affinis* 0.01 0.03 0.11 0.08 99.93

*Bathynectes* sp. 0.01 0.03 0.11 0.07 100.00

***Group j (Syr.Car)***

Average similarity: 37.04

Species Av.Abund Av.Sim Sim/SD Contrib% Cum.%

**Cnidaria sp. 1 0.14 16.12 1.32 43.53 43.53**

**Ophiuroidea sp. 8 0.09 8.70 1.20 23.48 67.01**

***Syringammina fragillissima* 0.06 5.95 1.14 16.06 83.07**

***Ophiactis abyssicola* 0.06 3.46 0.66 9.35 92.42**

Amphiuridae sp. 1 0.02 0.40 0.10 1.09 93.50

Cerianthidae sp. 1 0.02 0.40 0.24 1.08 94.58

Ophiuroidea sp. 2 0.02 0.33 0.17 0.90 95.48

Porifera encrusting sp. 28 0.01 0.33 0.24 0.88 96.36

*Echinus acutus* 0.02 0.28 0.17 0.76 97.12

*Pennatula phosphorea* 0.01 0.26 0.17 0.69 97.80

Actiniaria sp. 20 0.01 0.18 0.17 0.49 98.29

*Caryophyllia* sp. 2 0.02 0.17 0.10 0.47 98.76

Holothuroidea sp. 4 0.01 0.09 0.10 0.25 99.01

*Ophiactis balli* 0.02 0.09 0.10 0.25 99.26

Paguridae spp. 0.01 0.08 0.10 0.21 99.47

Porifera encrusting sp. 25 0.00 0.05 0.10 0.13 99.60

Benthogone sp. 0.01 0.05 0.10 0.13 99.72

Holothuroidea sp. 3 0.01 0.05 0.10 0.13 99.85

Porifera encrusting sp. 20 0.00 0.03 0.10 0.08 99.93

Porifera encrusting sp. 1 0.00 0.03 0.10 0.07 100.00

***Group k (Syr.Oph)***

Average similarity: 21.93

Species Av.Abund Av.Sim Sim/SD Contrib% Cum.%

***Syringammina fragillissima* 0.09 11.84 0.96 53.98 53.98**

**Porifera encrusting sp. 1 0.03 3.78 0.77 17.24 71.22**

**Ophiuroidea sp. 1 0.03 2.21 0.33 10.08 81.30**

**Porifera massive globose sp. 12 0.04 1.14 0.28 5.21 86.51**

Ophiuroidea sp. 2 0.03 0.54 0.20 2.46 88.98

*Lanice* sp. 1 0.02 0.51 0.21 2.31 91.28

Porifera encrusting sp. 28 0.01 0.37 0.29 1.70 92.98

*Psolus squamatus* 0.02 0.28 0.16 1.27 94.26

Porifera branching-erect sp. 2 0.02 0.20 0.11 0.90 95.15

*Colus* sp. 2 0.01 0.16 0.11 0.75 95.90

Serpulidae sp. 1 0.02 0.12 0.11 0.57 96.47

Porifera encrusting sp. 22 0.02 0.12 0.11 0.56 97.03

Porifera encrusting sp. 40 0.01 0.12 0.16 0.56 97.59

*Caryophyllia* sp. 2 0.01 0.08 0.07 0.39 97.98

Ophiuroidea sp. 8 0.01 0.08 0.07 0.34 98.32

Actiniaria sp. 4 0.01 0.07 0.07 0.34 98.66

Serpulidae sp. 2 0.01 0.07 0.07 0.31 98.97

Porifera encrusting sp. 3 0.01 0.06 0.07 0.26 99.23

Porifera massive globose sp. 4 0.01 0.04 0.07 0.20 99.43

Halcampoididae sp. 1 0.01 0.04 0.07 0.20 99.64

Holothuroidea sp. 4 0.01 0.04 0.07 0.17 99.80

Porifera encrusting sp. 25 0.00 0.04 0.11 0.16 99.96

Porifera encrusting sp. 31 0.00 0.01 0.07 0.04 100.00

***Group l***

Average similarity: 32.42

Species Av.Abund Av.Sim Sim/SD Contrib% Cum.%

**Porifera encrusting sp. 41 0.08 11.65 2.16 35.94 35.94**

***Psolus squamatus* 0.05 4.39 1.06 13.53 49.46**

**Porifera encrusting sp. 3 0.07 2.95 0.82 9.11 58.57**

***Madrepora oculata* 0.03 2.85 0.89 8.79 67.36**

***Solenosmilia variabilis* 0.04 2.58 0.93 7.96 75.31**

**Porifera encrusting sp. 22 0.03 2.57 0.57 7.94 83.25**

***Lophelia pertusa* (dead structure) 0.02 2.46 0.90 7.60 90.86**

**Porifera encrusting sp. 10 0.02 1.65 0.52 5.10 95.96**

Actiniaria sp. 0.02 0.51 0.32 1.58 97.54

*Stichopathes cf. gravieri* 0.02 0.51 0.32 1.58 99.12

Porifera encrusting sp. 1 0.01 0.28 0.32 0.88 100.00

***Group m***

Average similarity: 22.28

Species Av.Abund Av.Sim Sim/SD Contrib% Cum.%

**Porifera encrusting sp. 10 0.28 18.79 ####### 84.32 84.32**

**Porifera encrusting sp. 1 0.05 3.49 ####### 15.68 100.00**

***Group n***

Average similarity: 28.84

Species Av.Abund Av.Sim Sim/SD Contrib% Cum.%

**Porifera encrusting sp. 22**  **0.07 7.42 0.90 25.72 25.72**

**Porifera lamellate sp. 7 0.04 4.36 0.89 15.13 40.85**

***Lophelia pertusa* (dead structure) 0.04 3.57 0.84 12.36 53.21**

***Stichopathes cf. gravieri* 0.03 3.12 0.91 10.83 64.04**

**Crinoidea sp. 8 0.03 3.02 0.91 10.47 74.52**

***Henricia sanguinolenta* 0.03 2.82 0.91 9.78 84.29**

***Caryophyllia* sp. 2 0.03 1.91 0.41 6.61 90.90**

*Ceramaster/Peltaster/Plinthaster* 0.02 1.02 0.41 3.55 94.45

Porifera encrusting sp. 10 0.02 0.89 0.41 3.08 97.53

*Stichopathes* sp. 0.02 0.71 0.41 2.47 100.00

***Group o (Por.Pso)***

Average similarity: 36.27

Species Av.Abund Av.Sim Sim/SD Contrib% Cum.%

***Psolus squamatus* 0.27 21.34 2.32 58.84 58.84**

***Ophiactis balli* 0.08 2.59 0.59 7.13 65.97**

Porifera encrusting sp. 22 0.04 1.78 0.82 4.91 70.88

Porifera encrusting sp. 28 0.03 1.36 0.85 3.76 74.64

Porifera encrusting sp. 10 0.03 1.22 0.69 3.37 78.01

Caryophyllia sp. 2 0.03 1.19 0.56 3.27 81.28

Porifera encrusting sp. 1 0.03 0.95 0.58 2.62 83.91

*Lophelia pertusa* (dead structure) 0.02 0.64 0.48 1.76 85.67

Porifera encrusting sp. 41 0.02 0.49 0.38 1.35 87.02

Ascidiacea sp. 2 0.02 0.45 0.31 1.24 88.26

*Ophiactis abyssicola* 0.03 0.45 0.26 1.23 89.49

*Cidaris cidaris* 0.01 0.35 0.32 0.97 90.46

*Syringammina fragillissima* 0.01 0.34 0.28 0.94 91.41

Ophiuroidea sp. 2 0.01 0.34 0.29 0.93 92.34

Porifera massive lobose sp. 12 0.01 0.29 0.33 0.79 93.13

Porifera lamellate sp. 7 0.02 0.24 0.24 0.68 93.81

Porifera encrusting sp. 4 0.01 0.20 0.27 0.56 94.37

Serpulidae sp. 1 0.01 0.20 0.17 0.54 94.91

*Munida sarsi* 0.01 0.19 0.24 0.53 95.44

Porifera encrusting sp. 25 0.01 0.17 0.23 0.47 95.91

Porifera encrusting sp. 3 0.01 0.17 0.22 0.46 96.37

Porifera encrusting sp. 40 0.01 0.15 0.20 0.40 96.78

Actiniaria sp. 20 0.01 0.13 0.17 0.37 97.15

Porifera encrusting sp. 6 0.01 0.11 0.18 0.30 97.45

Cerianthidae sp. 1 0.01 0.10 0.17 0.28 97.73

*Ceramaster/Peltaster/Plinthaster* 0.01 0.09 0.15 0.25 97.97

Crinoidea sp. 1 0.01 0.07 0.15 0.21 98.18

Galatheidae sp. 1 0.01 0.07 0.13 0.20 98.38

*Henricia sanguinolenta* 0.01 0.06 0.12 0.17 98.54

*Pentametrocrinus atlanticus* 0.01 0.06 0.13 0.15 98.70

*Stichopathes* sp. 0.01 0.04 0.10 0.12 98.82

Actiniaria sp. 0.00 0.04 0.10 0.11 98.93

*Stichopathes cf. gravieri* 0.00 0.03 0.08 0.08 99.01

Holothuroidea sp. 3 0.00 0.03 0.08 0.08 99.09

Ascidiacea sp. 1 0.00 0.03 0.08 0.08 99.16

Porifera encrusting sp. 20 0.00 0.03 0.09 0.07 99.24

Porifera massive lobose sp. 20 0.01 0.03 0.10 0.07 99.31

Porifera encrusting sp. 2 0.00 0.02 0.07 0.06 99.37

Solenosmilia variabilis 0.00 0.02 0.09 0.06 99.43

Serpulidae sp. 2 0.01 0.02 0.08 0.06 99.49

Porifera encrusting sp. 15 0.00 0.02 0.10 0.06 99.54

Actiniaria sp. 9 0.00 0.02 0.08 0.05 99.60

Halcampoididae sp. 1 0.00 0.02 0.06 0.05 99.65

Porifera massive lobose sp. 19 0.00 0.02 0.06 0.05 99.69

*Stylaster* sp. 1 0.00 0.01 0.06 0.04 99.73

Porifera encrusting sp. 16 0.00 0.01 0.05 0.03 99.76

Porifera branching-erect sp. 2 0.00 0.01 0.05 0.03 99.79

Porifera encrusting sp. 8 0.00 0.01 0.08 0.03 99.82

Brachiopoda sp. 1 0.00 0.01 0.06 0.03 99.85

Halcampoididae sp. 3 0.01 0.01 0.05 0.03 99.88

Hydrozoa (bushy) 0.01 0.01 0.04 0.02 99.90

Porifera massive lobose sp. 18 0.00 0.01 0.03 0.02 99.92

*Pandalus borealis* 0.00 0.00 0.03 0.01 99.93

Porifera massive lobose sp. 13 0.00 0.00 0.03 0.01 99.94

*Parantipathes* sp.2 0.00 0.00 0.03 0.01 99.95

*Parantipathes* sp.1 0.00 0.00 0.03 0.01 99.96

Ascidiacea sp. 5 0.00 0.00 0.03 0.01 99.97

Porifera massive globose sp. 4 0.00 0.00 0.03 0.01 99.97

Decapoda sp. 5 0.00 0.00 0.03 0.01 99.98

*Margarites* sp. 1 0.00 0.00 0.03 0.01 99.99

Majidae sp. 1 0.01 0.00 0.03 0.01 100.00

***Group p (Ser.Pso)***

Average similarity: 25.97

Species Av.Abund Av.Sim Sim/SD Contrib% Cum.%

***Ophiactis abyssicola* 0.12 4.03 0.85 15.50 15.50**

**Ophiuroidea sp. 6 0.09 2.99 0.63 11.53 27.03**

***Ophiactis balli* 0.09 2.35 0.62 9.03 36.06**

**Serpulidae sp. 1 0.05 2.08 0.57 8.02 44.08**

**Majidae sp. 1 0.08 2.05 0.58 7.88 51.96**

***Psolus squamatus* 0.06 1.93 0.64 7.43 59.39**

**Porifera encrusting sp. 1 0.04 1.44 0.80 5.55 64.94**

Porifera encrusting sp. 28 0.03 1.22 0.81 4.68 69.62

Porifera encrusting sp. 6 0.03 0.96 0.47 3.68 73.31

Ascidiacea sp. 2 0.04 0.81 0.41 3.11 76.42

*Lophelia pertusa* (dead structure) 0.04 0.80 0.45 3.09 79.51

Porifera massive lobose sp. 20 0.04 0.76 0.38 2.94 82.45

Porifera encrusting sp. 10 0.03 0.58 0.45 2.24 84.70

Ophiuroidea sp. 2 0.03 0.46 0.29 1.78 86.48

Porifera massive globose sp. 12 0.02 0.46 0.28 1.78 88.26

Porifera massive globose sp. 4 0.03 0.32 0.28 1.24 89.50

Serpulidae sp. 2 0.03 0.31 0.21 1.21 90.71

*Reteporella* sp. 2 0.02 0.29 0.25 1.11 91.81

Porifera encrusting sp. 3 0.02 0.28 0.36 1.08 92.89

*Syringammina fragillissima* 0.02 0.21 0.18 0.80 93.70

Porifera encrusting sp. 22 0.02 0.21 0.26 0.79 94.49

Ascidiacea sp. 5 0.02 0.19 0.21 0.72 95.21

Porifera encrusting sp. 42 0.01 0.14 0.21 0.54 95.74

Porifera massive lobose sp. 19 0.02 0.12 0.17 0.48 96.22

Porifera encrusting sp. 31 0.01 0.10 0.16 0.39 96.61

Porifera encrusting sp. 25 0.01 0.08 0.19 0.31 96.92

*Lophelia pertusa* 0.01 0.07 0.17 0.27 97.19

Gastropoda sp. 1 0.01 0.06 0.10 0.23 97.42

Actiniaria sp. 13 0.01 0.06 0.09 0.23 97.65

Halcampoididae sp. 1 0.01 0.06 0.13 0.23 97.88

Porifera massive globose sp. 7 0.01 0.05 0.09 0.20 98.07

Zoanthidea sp. 2 0.01 0.05 0.12 0.19 98.27

Porifera massive lobose sp. 12 0.01 0.05 0.12 0.19 98.45

*Cidaris cidaris* 0.01 0.05 0.14 0.18 98.63

Porifera encrusting sp. 41 0.02 0.05 0.13 0.18 98.81

*Stylaster* sp. 1 0.01 0.04 0.13 0.15 98.97

Crinoidea sp. 5 0.01 0.03 0.10 0.11 99.08

Crinoidea sp. 1 0.01 0.03 0.09 0.11 99.18

*Caryophyllia* sp. 2 0.01 0.03 0.09 0.10 99.28

Ophiuroidea sp. 8 0.01 0.02 0.06 0.09 99.37

Porifera encrusting sp. 26 0.00 0.02 0.09 0.08 99.45

Decapoda sp. 2 0.01 0.02 0.06 0.08 99.52

Porifera encrusting sp. 20 0.00 0.02 0.10 0.07 99.60

Actiniaria sp. 5 0.01 0.02 0.06 0.06 99.66

Porifera encrusting sp. 9 0.01 0.01 0.06 0.05 99.72

Porifera encrusting sp. 4 0.00 0.01 0.10 0.05 99.77

*Henricia sanguinolenta* 0.00 0.01 0.06 0.04 99.80

Margarites sp. 1 0.01 0.01 0.06 0.03 99.84

Corallimorphidae sp. 2 0.00 0.01 0.06 0.03 99.87

*Antipatharia* sp. 4 0.00 0.01 0.06 0.03 99.90

Caryophyllia sp. 3 0.01 0.01 0.06 0.03 99.93

Porifera encrusting sp. 15 0.01 0.01 0.06 0.02 99.95

Ophiuroidea sp. 7 0.01 0.01 0.06 0.02 99.98

Porifera encrusting sp. 40 0.00 0.00 0.06 0.01 99.99

Hydrozoa (bushy) 0.00 0.00 0.06 0.01 100.00

***Group q***

Average similarity: 35.04

Species Av.Abund Av.Sim Sim/SD Contrib% Cum.%

***Ophiactis balli* 0.08 15.04 1.50 42.91 42.91**

***Caryophyllia* sp. 2 0.06 11.28 1.41 32.19 75.11**

Porifera encrusting sp. 1 0.01 1.34 0.65 3.83 78.94

Cnidaria sp. 1 0.02 1.24 0.33 3.53 82.47

Porifera encrusting sp. 22 0.01 1.13 0.68 3.22 85.70

Ophiuroidea sp. 2 0.02 1.08 0.33 3.08 88.78

*Lophelia pertusa* (dead structure) 0.02 0.85 0.34 2.44 91.22

Porifera encrusting sp. 28 0.02 0.78 0.30 2.21 93.43

Porifera encrusting sp. 6 0.01 0.56 0.34 1.59 95.02

Ascidiacea sp. 2 0.01 0.48 0.19 1.38 96.40

Porifera encrusting sp. 25 0.02 0.39 0.30 1.10 97.50

Actiniaria sp. 20 0.01 0.31 0.19 0.90 98.40

*Pandalus borealis* 0.01 0.31 0.19 0.89 99.29

*Pentametrocrinus atlanticus* 0.01 0.25 0.19 0.71 100.00

***Group r***

Average similarity: 34.84

Species Av.Abund Av.Sim Sim/SD Contrib% Cum.%

**Ophiuroidea sp. 8 0.12 12.38 1.74 35.52 35.52**

***Caryophyllia* sp. 2 0.06 6.21 2.58 17.82 53.34**

**Ophiuroidea sp. 2 0.05 4.61 0.97 13.23 66.58**

***Pandalus borealis* 0.04 3.05 0.93 8.75 75.33**

Porifera encrusting sp. 10 0.03 1.72 0.45 4.93 80.26

*Stichopathes cf. gravieri* 0.04 1.44 0.47 4.12 84.38

*Ophiomusium lymani* 0.02 0.85 0.34 2.44 86.82

Ophiuroidea sp. 1 0.02 0.57 0.34 1.63 88.46

Porifera encrusting sp. 6 0.02 0.57 0.66 1.63 90.08

Crinoidea sp. 8 0.02 0.53 0.34 1.52 91.61

Porifera encrusting sp. 22 0.06 0.45 0.40 1.30 92.91

Ascidiacea sp. 2 0.01 0.42 0.33 1.20 94.11

Porifera encrusting sp. 28 0.02 0.33 0.33 0.96 95.07

Porifera encrusting sp. 1 0.01 0.27 0.19 0.76 95.83

*Psolus squamatus* 0.01 0.21 0.19 0.60 96.43

Cerianthidae sp. 1 0.01 0.19 0.19 0.55 96.98

Porifera massive lobose sp. 16 0.01 0.19 0.19 0.55 97.53

*Echinus* spp. 0.01 0.18 0.19 0.53 98.06

*Syringammina fragillissima* 0.01 0.18 0.19 0.53 98.59

*Echinus acutus* 0.01 0.17 0.19 0.50 99.09

Actiniaria sp. 9 0.01 0.12 0.19 0.33 99.43

Porifera boring sp. 1 0.01 0.11 0.19 0.31 99.74

*Lophelia pertusa* (dead structure) 0.01 0.05 0.19 0.15 99.89

Porifera encrusting sp.40 0.02 0.04 0.19 0.11 100.00

***Group s***

Less than 2 samples in group

***Group t (Sol.Oph)***

Average similarity: 34.24

Species Av.Abund Av.Sim Sim/SD Contrib% Cum.%

***Ophiactis balli* 0.35 18.58 2.66 54.26 54.26**

***Ophiactis abyssicola* 0.13 3.00 0.73 8.75 63.01**

**Porifera encrusting sp. 42 0.11 2.04 0.53 5.97 68.98**

***Solenosmila variabilis* (dead structure) 0.08 1.86 0.88 5.42 74.40**

Ascidiacea sp. 2 0.05 1.30 0.82 3.81 78.21

Porifera encrusting sp. 6 0.05 1.21 0.48 3.52 81.73

Ophiuroidea sp. 2 0.07 1.07 0.40 3.11 84.84

*Solenosmilia variabilis* 0.07 0.79 0.36 2.32 87.16

Crinoidea sp. 8 0.03 0.53 0.45 1.56 88.72

Caryophyllia sp. 2 0.02 0.47 0.43 1.38 90.09

Porifera encrusting sp. 1 0.03 0.39 0.20 1.14 91.23

*Psolus squamatus* 0.02 0.38 0.35 1.11 92.34

Decapoda sp. 5 0.02 0.28 0.37 0.82 93.16

Porifera massive lobose sp. 12 0.02 0.21 0.35 0.61 93.78

Porifera encrusting sp. 10 0.02 0.20 0.31 0.59 94.37

Porifera encrusting sp. 39 0.02 0.19 0.29 0.54 94.91

Porifera encrusting sp. 28 0.01 0.18 0.24 0.54 95.45

Isididae sp. 2 0.01 0.15 0.26 0.43 95.89

*Syringammina fragillissima* 0.02 0.13 0.17 0.39 96.28

Porifera encrusting sp. 22 0.01 0.12 0.17 0.34 96.62

Ophiuroidea sp. 8 0.02 0.11 0.13 0.33 96.95

*Anthothela grandiflora* 0.05 0.11 0.08 0.31 97.26

Crinoidea sp. 1 0.01 0.07 0.19 0.21 97.47

*Cidaris cidaris* 0.01 0.07 0.14 0.21 97.68

*Stichopathes cf_ gravieri* 0.01 0.07 0.15 0.19 97.87

Porifera encrusting sp. 15 0.01 0.07 0.16 0.19 98.07

*Pandalus borealis* 0.01 0.06 0.15 0.18 98.24

*Stichopathes* sp. 0.01 0.06 0.14 0.17 98.42

Porifera massive lobose sp. 21 0.03 0.05 0.05 0.15 98.57

Porifera massive lobose sp. 2 0.03 0.05 0.11 0.14 98.71

*Echinus* spp. 0.01 0.05 0.15 0.14 98.85

*Koehlermetra porrecta* 0.01 0.05 0.15 0.13 98.99

Porifera massive lobose sp. 18 0.01 0.03 0.17 0.10 99.08

Porifera encrusting sp.25 0.00 0.03 0.13 0.08 99.17

Actiniaria sp.20 0.01 0.03 0.08 0.07 99.24

Sabellidae sp. 2 0.02 0.02 0.05 0.06 99.30

Porifera lamellate sp. 7 0.00 0.02 0.08 0.05 99.35

Halcampoididae sp. 1 0.01 0.02 0.08 0.05 99.40

Serpulidae sp. 1 0.00 0.02 0.08 0.05 99.45

Cerianthidae sp. 1 0.01 0.02 0.08 0.05 99.49

Actiniaria sp. 0.00 0.02 0.08 0.05 99.54

Actiniaria sp. 6 0.00 0.02 0.08 0.04 99.58

*Pentametrocrinus atlanticus* 0.00 0.02 0.08 0.04 99.63

*Henricia sanguinolenta* 0.01 0.01 0.08 0.04 99.67

Porifera encrusting sp. 3 0.00 0.01 0.11 0.04 99.71

Porifera massive globose sp. 4 0.00 0.01 0.08 0.04 99.75

*Brisingella coronata / Brisinga endecacnemos* 0.00 0.01 0.08 0.04 99.79

Ophiuroidea sp. 9 0.01 0.01 0.05 0.04 99.82

Zoanthidae sp.6 0.00 0.01 0.08 0.02 99.85

Galatheidae sp.1 0.00 0.01 0.05 0.02 99.86

*Acanthogorgia armarta* 0.00 0.01 0.05 0.02 99.88

*Munida sarsi* 0.00 0.01 0.05 0.02 99.89

Ophiuroidea sp.7 0.00 0.01 0.05 0.02 99.91

*Margarites* sp.1 0.00 0.01 0.05 0.02 99.92

*Parantipathes* sp.1 0.00 0.00 0.05 0.01 99.94

Isididae sp. 1 0.00 0.00 0.05 0.01 99.95

*Anthomastus grandiflora* 0.00 0.00 0.05 0.01 99.97

Ascidiacea sp. 1 0.00 0.00 0.05 0.01 99.98

*Echinus acutus* 0.00 0.00 0.05 0.01 99.99

Porifera encrusting sp.16 0.00 0.00 0.05 0.01 100.00

***Group u (Lop.Oph)***

Average similarity: 24.71

Species Av.Abund Av.Sim Sim/SD Contrib% Cum.%

***Lophelia pertusa* 0.13 4.72 0.72 19.11 19.11**

***Ophiactis balli* 0.18 4.39 0.53 17.77 36.88**

***Lophelia pertusa* (dead structure) 0.05 2.40 0.86 9.71 46.59**

***Cidaris cidaris* 0.06 2.15 0.84 8.70 55.29**

***Madrepora oculata* 0.08 2.09 0.49 8.47 63.76**

**Actiniaria sp. 0.07 1.66 0.60 6.73 70.49**

***Protanthea simple* 0.07 1.33 0.40 5.40 75.89**

Ascidiacea sp. 2 0.03 1.05 0.61 4.24 80.13

Cerianthidae sp.1 0.04 0.93 0.61 3.75 83.88

Actiniaria sp. 9 0.04 0.83 0.39 3.34 87.22

Psolus squamatus 0.04 0.81 0.40 3.27 90.49

Decapoda sp.5 0.02 0.66 0.37 2.67 93.15

Porifera encrusting sp.10 0.02 0.25 0.37 1.03 94.18

Porifera encrusting sp.42 0.03 0.21 0.22 0.85 95.03

Ophiuroidea sp. 2 0.02 0.17 0.22 0.68 95.71

Serpulidae sp. 1 0.02 0.17 0.22 0.68 96.39

Hydrozoa (flat branched) 0.02 0.15 0.22 0.60 96.99

Porifera lamellate sp. 7 0.01 0.15 0.22 0.60 97.59

Crinoidea sp. 1 0.01 0.14 0.22 0.56 98.15

Bathynectes sp_ 0.02 0.12 0.22 0.47 98.62

Corallimorphidae sp_ 2 0.03 0.12 0.22 0.47 99.09

Porifera encrusting sp_ 1 0.01 0.11 0.22 0.43 99.52

Porifera encrusting sp_ 6 0.01 0.08 0.22 0.32 99.84

Hydrozoa (bushy) 0.01 0.04 0.22 0.16 100.00

***Group v***

Less than 2 samples in group

***Group w (Sol.Car)***

Average similarity: 40.96

Species Av.Abund Av.Sim Sim/SD Contrib% Cum.%

**Ophiuroidea sp. 2 0.18 9.11 1.54 22.25 22.25**

**Ophiuroidea sp. 8 0.13 7.36 2.25 17.97 40.22**

**Porifera encrusting sp. 6 0.11 3.22 0.69 7.87 48.08**

***Caryophyllia* sp. 2 0.06 2.80 1.34 6.84 54.93**

**Porifera encrusting sp. 39 0.08 2.80 0.84 6.84 61.76**

**Crinoidea sp. 1 0.05 2.68 1.42 6.55 68.31**

***Solenosmilia variabilis* (dead structure) 0.07 2.45 0.83 5.99 74.30**

*Ophiactis balli* 0.07 2.01 0.54 4.90 79.20

*Solenosmilia variabilis* 0.05 1.53 0.73 3.74 82.95

*Psolus squamatus* 0.04 1.51 0.96 3.69 86.63

Porifera encrusting sp. 10 0.02 0.77 0.58 1.87 88.50

Porifera encrusting sp. 22 0.03 0.57 0.32 1.39 89.89

Porifera encrusting sp. 28 0.02 0.45 0.54 1.09 90.98

Porifera encrusting sp. 1 0.02 0.42 0.42 1.02 92.00

*Stichopathes cf. gravieri* 0.02 0.41 0.37 1.01 93.01

Ascidiacea sp. 2 0.02 0.38 0.39 0.92 93.93

*Ophiactis abyssicola* 0.02 0.35 0.31 0.85 94.79

Porifera massive lobose sp. 12 0.02 0.30 0.24 0.73 95.52

Crinoidea sp. 9 0.01 0.30 0.32 0.72 96.24

*Echinus acutus* 0.01 0.24 0.33 0.59 96.84

Actiniaria sp. 0.01 0.21 0.27 0.50 97.34

Isididae sp. 2 0.01 0.14 0.22 0.33 97.67

*Pandalus borealis* 0.01 0.13 0.22 0.31 97.98

Porifera encrusting sp. 42 0.01 0.12 0.22 0.30 98.28

*Koehlermetra porrecta* 0.01 0.12 0.22 0.28 98.56

*Brisingella coronata* / *Brisinga endecacnemos* 0.01 0.08 0.17 0.20 98.76

Ascidiacea sp. 1 0.01 0.08 0.17 0.19 98.95

Crinoidea sp. 8 0.01 0.07 0.17 0.16 99.12

*Ceramaster/Peltaster/Plinthaster* 0.01 0.06 0.17 0.16 99.27

Porifera massive lobose sp. 18 0.01 0.06 0.16 0.15 99.42

*Pentametrocrinus atlanticus* 0.01 0.04 0.12 0.10 99.52

Actiniaria sp. 6 0.01 0.04 0.12 0.09 99.61

*Bathypathes* sp.2 0.00 0.03 0.12 0.07 99.68

*Parantipathes* sp.2 0.01 0.02 0.07 0.05 99.72

Decapoda sp. 5 0.00 0.01 0.07 0.03 99.75

Ophiuroidea sp. 1 0.00 0.01 0.07 0.03 99.79

*Stichastrella rosea* 0.00 0.01 0.07 0.03 99.82

*Henricia sanguinolenta* 0.00 0.01 0.07 0.03 99.84

Cerianthidae sp. 1 0.00 0.01 0.07 0.03 99.87

*Echinus* spp. 0.00 0.01 0.07 0.02 99.89

*Anthomastus grandiflora* 0.00 0.01 0.07 0.02 99.92

Ascidiacea sp. 4 0.00 0.01 0.07 0.02 99.94

Porifera encrusting sp. 40 0.00 0.01 0.07 0.02 99.96

Porifera encrusting sp. 25 0.00 0.01 0.07 0.02 99.98

Porifera encrusting sp. 2 0.00 0.01 0.07 0.02 100.00

***Group x (Sol.Por)***

Average similarity: 39.80

Species Av.Abund Av.Sim Sim/SD Contrib% Cum.%

**Ophiuroidea sp. 2 0.19 8.33 2.03 20.94 20.94**

***Ophiactis abyssicola* 0.19 6.56 1.51 16.48 37.42**

***Ophiactis balli* 0.14 5.26 0.89 12.21 49.63**

**Ophiuroidea sp. 8 0.11 3.96 1.04 8.96 59.59**

**Porifera encrusting sp. 39 0.07 1.78 0.93 5.48 65.07**

***Solenosmilia variabilis* (dead structure) 0.06 1.77 1.13 5.45 69.52**

Porifera encrusting sp. 6 0.04 1.56 1.16 3.92 73.44

Porifera encrusting sp. 10 0.05 1.15 0.61 2.89 76.33

*Caryophyllia* sp. 2 0.04 1.11 0.70 2.79 79.12

Crinoidea sp. 8 0.05 1.05 0.65 2.64 81.76

Porifera encrusting sp. 42 0.03 0.82 0.74 2.07 83.83

Porifera encrusting sp. 25 0.05 0.82 0.48 2.07 85.90

Porifera encrusting sp. 28 0.06 0.82 0.47 2.05 87.95

Porifera massive lobose sp. 18 0.05 0.72 0.32 1.80 89.76

*Syringammina fragillissima* 0.03 0.57 0.41 1.43 91.18

*Psolus squamatus* 0.03 0.51 0.38 1.28 92.46

*Pandalus borealis*  0.02 0.43 0.40 1.08 93.54

Porifera encrusting sp. 22 0.03 0.40 0.39 1.01 94.55

Ascidiacea sp. 2 0.02 0.33 0.32 0.84 95.39

Ascidiacea sp. 1 0.03 0.32 0.31 0.82 96.20

Porifera encrusting sp. 1 0.02 0.32 0.47 0.80 97.00

*Solenosmilia variabilis* 0.01 0.19 0.33 0.49 97.49

Serpulidae sp. 1 0.02 0.10 0.17 0.26 97.75

*Echinus* spp. 0.01 0.08 0.18 0.21 97.95

Crinoidea sp. 9 0.01 0.08 0.18 0.19 98.15

*Echinus acutus* 0.01 0.07 0.15 0.17 98.32

Decapoda sp. 5 0.01 0.07 0.18 0.17 98.49

*Anthomastus grandiflora* 0.01 0.06 0.15 0.16 98.65

Porifera encrusting sp. 3 0.01 0.06 0.16 0.15 98.80

Halcampoididae sp. 1 0.01 0.06 0.15 0.14 98.94

Cnidaria sp. 1 0.01 0.04 0.10 0.11 99.05

Ophiuroidea sp. 1 0.01 0.04 0.13 0.10 99.16

Isididae sp. 2 0.01 0.04 0.13 0.10 99.25

Crinoidea sp. 1 0.01 0.03 0.12 0.09 99.34

Porifera encrusting sp. 40 0.01 0.03 0.10 0.08 99.42

*Reteporella* sp. 2 0.01 0.03 0.09 0.08 99.49

Actiniaria sp. 0.01 0.03 0.10 0.07 99.57

Porifera massive lobose sp. 12 0.00 0.03 0.16 0.07 99.64

*Pentametrocrinus atlanticus* 0.01 0.02 0.07 0.04 99.68

Porifera encrusting sp. 2 0.01 0.02 0.08 0.04 99.72

*Ophiomusium lymani* 0.00 0.02 0.07 0.04 99.76

Porifera encrusting sp. 16 0.01 0.01 0.04 0.04 99.80

Porania pulvillus 0.01 0.01 0.07 0.03 99.83

Actiniaria sp. 9 0.00 0.01 0.07 0.03 99.87

*Jasonisis* sp. nov 0.00 0.01 0.07 0.03 99.89

Zoanthidae sp.6 0.00 0.01 0.07 0.02 99.92

Paguridae spp. 0.00 0.01 0.04 0.01 99.93

*Parantipathes* sp.2 0.00 0.00 0.04 0.01 99.94

Porifera massive globose sp. 4 0.00 0.00 0.04 0.01 99.95

*Keratoisis* sp. 2 0.00 0.00 0.04 0.01 99.96

Porifera massive globose sp. 12 0.00 0.00 0.04 0.01 99.97

*Brisingella coronata* / *Brisinga endecacnemos* 0.00 0.00 0.04 0.01 99.98

Ophiuroidea sp. 7 0.00 0.00 0.04 0.01 99.99

Cerianthidae sp. 1 0.00 0.00 0.04 0.01 100.00

***Group y (Lep.par)***

Average similarity: 28.61

Species Av.Abund Av.Sim Sim/SD Contrib% Cum.%

***Caryophyllia* sp. 2 0.05 12.48 2.94 43.61 43.61**

**Porifera encrusting sp. 28 0.05 4.21 0.48 14.71 58.32**

***Lepidisis* sp. 0.03 3.18 0.63 11.12 69.44**

***Psolus squamatus* 0.03 2.21 0.46 7.74 77.18**

***Parantipathes* sp.1 0.02 2.16 0.48 7.56 84.74**

*Keratoisis* sp. 2 0.01 0.94 0.32 3.28 88.01

Ophiuroidea sp. 2 0.04 0.71 0.19 2.48 90.50

Porifera massive lobose sp. 18 0.02 0.61 0.19 2.13 92.62

Porifera encrusting sp. 6 0.01 0.50 0.28 1.75 94.37

*Anthomastus grandiflora* 0.01 0.42 0.19 1.49 95.85

Actiniaria sp. 0.01 0.42 0.19 1.48 97.33

Porifera lamellate sp. 7 0.01 0.34 0.19 1.20 98.53

Porifera encrusting sp. 10 0.02 0.23 0.19 0.82 99.35

Crinoidea sp. 1 0.01 0.19 0.19 0.65 100.00

***Group z (Ker.Sol)***

Average similarity: 27.89

Species Av.Abund Av.Sim Sim/SD Contrib% Cum.%

***Solenosmilia variabilis* 0.06 8.68 1.41 31.12 31.12**

**Porifera encrusting sp. 6 0.03 5.19 0.67 18.60 49.72**

***Keratoisis* sp. 2 0.02 4.89 0.77 17.52 67.24**

***Caryophyllia* sp. 2 0.03 3.94 0.87 14.13 81.37**

**Ophiuroidea sp. 4 0.04 1.62 0.37 5.81 87.18**

*Koehlermetra porrecta* 0.01 0.76 0.26 2.73 89.91

*Lophelia pertusa* (dead structure) 0.01 0.65 0.37 2.33 92.25

*Lepidisis* sp. 0.01 0.62 0.26 2.21 94.45

Ophiuroidea sp. 2 0.02 0.36 0.15 1.29 95.74

Ascidiacea sp. 2 0.01 0.33 0.25 1.18 96.92

Ophiactis balli 0.01 0.20 0.15 0.71 97.63

Actiniaria sp. 6 0.01 0.20 0.15 0.70 98.34

Crinoidea sp. 1 0.01 0.12 0.15 0.44 98.78

*Aphrocallistes* sp. 0.01 0.11 0.15 0.39 99.17

*Brisingella coronata / Brisinga endecacnemos* 0.01 0.10 0.15 0.35 99.51

*Pandalus borealis* 0.01 0.09 0.15 0.31 99.82

Porifera encrusting sp. 10 0.01 0.05 0.15 0.18 100.00

***Group aa (Gor.Zoa)***

Average similarity: 28.14

Species Av.Abund Av.Sim Sim/SD Contrib% Cum.%

***Solenosmilia variabilis* 0.10 7.03 1.17 25.00 25.00**

***Ophiactis balli* 0.11 4.15 0.58 14.77 39.77**

***Lophelia pertusa* (dead structure) 0.07 3.72 0.82 13.22 52.99**

**Isididae sp. 2 0.04 2.47 0.84 8.76 61.75**

**Zoanthidae sp.6 0.06 2.41 0.60 8.57 70.32**

**Porifera encrusting sp. 6 0.05 1.70 0.47 6.03 76.35**

***Anthomastus grandiflora* 0.03 1.43 0.64 5.08 81.43**

Porifera encrusting sp. 42 0.05 1.38 0.51 4.92 86.35

Isididae sp. 3 0.02 0.60 0.29 2.12 88.47

Ophiuroidea sp. 2 0.04 0.49 0.31 1.73 90.19

Crinoidea sp. 8 0.02 0.36 0.32 1.29 91.48

*Jasonisis* sp. nov 0.01 0.36 0.28 1.27 92.75

Ascidiacea sp. 2 0.01 0.34 0.32 1.21 93.96

*Ophiactis abyssicola*  0.02 0.23 0.22 0.83 94.79

Porifera encrusting sp. 39 0.02 0.19 0.25 0.68 95.47

Pycnogonida sp. 2 0.01 0.19 0.23 0.67 96.14

*Caryophyllia* sp. 2 0.01 0.16 0.23 0.58 96.72

*Koehlermetra porrecta* 0.01 0.13 0.19 0.45 97.17

Hydrozoa (bushy) 0.02 0.12 0.17 0.43 97.60

*Leiopathes* sp. 1 0.00 0.10 0.08 0.37 97.97

Porifera encrusting sp. 10 0.01 0.07 0.17 0.23 98.20

Porifera massive lobose sp. 8 0.01 0.06 0.16 0.23 98.43

*Keratoisis* sp. 2 0.01 0.05 0.15 0.19 98.62

Keratoisidinae sp.1 0.00 0.05 0.09 0.19 98.81

Zoanthidea sp. 6 0.01 0.05 0.06 0.16 98.97

Crinoidea sp. 3 0.01 0.04 0.10 0.15 99.12

Actiniaria sp. 6 0.00 0.03 0.10 0.11 99.23

*Aphrocallistes* sp. 0.00 0.03 0.10 0.11 99.34

Gorgonacea 0.01 0.03 0.06 0.11 99.45

*Psolus squamatus* 0.00 0.03 0.10 0.10 99.54

Porifera encrusting sp. 25 0.00 0.02 0.10 0.08 99.63

*Echinus* spp. 0.00 0.02 0.10 0.08 99.70

*Brisingella coronata. Brisinga endecacnemos* 0.00 0.02 0.06 0.07 99.77

Porifera encrusting sp. 1 0.00 0.01 0.08 0.05 99.82

Lepidisis sp. 0.00 0.01 0.06 0.04 99.86

Porifera massive lobose sp. 12 0.00 0.01 0.06 0.04 99.90

*Ceramaster/Peltaster/Plinthaster* 0.00 0.01 0.06 0.03 99.93

Actiniaria sp. 13 0.00 0.01 0.06 0.03 99.95

Porifera massive lobose sp. 18 0.00 0.01 0.06 0.02 99.98

*Parantipathes* sp.1 0.00 0.01 0.06 0.02 100.00
