# Supplementary material for: Benthic Assemblages of the Anton Dohrn Seamount (NE Atlantic): Defining Deep-Sea Biotopes to Support Habitat Mapping and Management Efforts with a Focus on Vulnerable Marine Ecosystems
Source: PLoS One. 2015 May 18;10(5):e0124815. doi: 10.1371/journal.pone.0124815 (PMC4436255; doi:10.1371/journal.pone.0124815)
Supplement: S1 Table — Clusters identified using the SIMPROF routine, SIMPER similarity, environmental variables and characterising species for each cluster identified. (DOCX) [file pone.0124815.s002.docx]

**S1 Table: Results of multivariate analysis**. Clusters identified using the SIMPROF routine, SIMPER similarity, environmental variables and characterising species for each cluster identified.

| **Cluster** | **No. images** | **Useful mapping units** | **SIMPER similarity level (%)** | **Temp range (°C)** | **Average temp (SD)** | **Depth range (m)** | **Average depth (SD)** | **Characterising species** |
| --- | --- | --- | --- | --- | --- | --- | --- | --- |
| a | 1 | N |  |  | 3.8 |  | 1720 |  |
| b | 1 | N |  |  | 3.89 |  | 1735 |  |
| c | 1 | N |  |  | 3.76 |  | 1855.7 |  |
| d | 1 | N |  |  | 5.5 |  | 1314.6 |  |
| e | 1 | N |  |  | 5 |  | 1520.6 |  |
| f | 1 | N |  |  | 7.8 |  | 1087.5 |  |
| g | 16 | Y | 21.35 | 3.69-3.84 | 3.74 (0.05) | 1794-1887 | 1849.9 (36.7) | *Ophiomusium lymani,* Unknown sp. 29, Crinoidea sp. 7 |
| h | 17 | N | 14.51 | 3.68-9 | 7.43 (1.78) | 810-1883 | 1089.8 (315.1) | Porifera encrusting sp. 1, Serpulidae sp. 1, Actiniaria sp. 9 |
| i | 9 | Y | 35.22 | 8.8-9.1 | 8.94 (0.08) | 749-794 | 771.9 (14.6) | *Madrepora oculata*,  *Lophelia pertusa* (dead structure), Decapoda sp. 5, *Cidaris cidaris*,  *Lophelia pertusa*, Actiniaria sp. |
| j | 15 | Y | 37.04 | 3.77-3.9 | 3.81 (0.03) | 1717-1822 | 1762.7 (23.2) | Cnidaria sp. 1, Ophiuroidea sp. 8,  *Syringammina fragilissima*, *Ophiactis abyssicola* |
| k | 22 | Y | 21.93 | 3.7-9 | 5.78 (1.49) | 818-1885 | 1364.9 (270) | *Syringammina fragilissima*, Porifera encrusting sp. 1, Ophiuroidea sp. 1, Porifera massive globose sp. 12 |
| l | 5 | N | 32.42 | 8.8-9 | 8.9 (0.06) | 854-987 | 910.6 (48) | Porifera encrusting sp. 41,  *Psolus squamatus*, Porifera encrusting sp. 3,  *Madrepora oculata*,  *Solenosmilia variabilis*, Porifera encrusting sp. 22,  *Lophelia pertusa* (dead structure), Porifera encrusting sp. 10 |
| m | 2 | N | 22.28 | 5.5-6.8 | 6.15 (0.9) | 1152-1317 | 1235.1 (116.5) | Porifera encrusting sp. 10, Porifera encrusting sp. 1 |
| n | 4 | N | 28.84 | 5.8-8.17 | 6.49 (1.12) | 1037-1266 | 1194.8 (106.1) | Porifera encrusting sp. 22, Porifera lamellate sp. 7,  *Solenosmilia variabilis* (dead structure),  *Stichopathes cf. Gravieri*, Crinoidea sp. 8,  *Henricia sanguinolenta*, *Caryophyllia* sp. 2 |
| o | 44 | Y | 36.27 | 5.2-9 | 7.06 (1.03) | 850-1352 | 1135 (116.7) | *Psolus squamatus*,  *Ophiactis balli* |
| p | 26 | Y | 25.97 | 4.4-8.8 | 7.9 (0.83) | 852-1529 | 1024.8 (116.6) | *Ophiactis abyssicola*, Ophiuroidea sp. 6,  *Ophiactis balli*, Serpulidae sp. 1, Majidae sp. 1,  *Psolus squamatus*, Porifera encrusting sp. 1 |
| q | 7 | N | 35.04 | 3.73-5.38 | 5.02 (0.57) | 1331-1803 | 1439.5 (162.5) | *Ophiactis balli*,  *Caryophyllia* sp. 2 |
| r | 7 | N | 34.84 | 3.76-4.69 | 4.27 (0.41) | 1501-1877 | 1644.7 (153.5) | Ophiuroidea sp. 8, *Caryophyllia* sp. 2, Ophiuroidea sp. 2,  *Pandalus borealis* |
| s | 1 | N |  |  | 5.6 |  | 1303.3 |  |
| t | 30 | Y | 33.24 | 4-6.86 | 5.29 (0.61) | 1140-1643 | 1381.7 (108.9) | *Ophiactis balli*,  *Ophiactis abyssicola*, Porifera encrusting sp. 42,  *Solenosmilia variabilis* (dead structure) |
| u | 8 | Y | 24.71 | 8.1-9.1 | 8.78 (0.39) | 766-991 | 840.7 (99) | *Lophelia pertusa*,  *Ophiactis balli*, *Lophelia pertusa* (dead structure),  *Cidaris cidaris*, *Madrepora oculata ,*  Actiniaria sp.,  *Protanthea simple* |
| v | 2 | N |  |  | 5.2 |  | 1340.6 |  |
| w | 21 | Y | 40.96 | 3.8-4.75 | 4.36 (0.27) | 1508-1736 | 1591.2 (67) | Ophiuroidea sp. 2, Ophiuroidea sp. 8, Porifera encrusting sp. 6,  *Caryophyllia* sp. 2, Porifera encrusting sp. 39, Crinoidea sp. 1,  *Solenosmilia variabilis* (dead structure) |
| x | 36 | Y | 39.80 | 3.8-5.3 | 4.16 (0.34) | 1342-1768 | 1641.4 (108) | Ophiuroidea sp. 2, *Ophiactis abyssicola*,  *Ophiactis balli*, Ophiuroidea sp. 8 |
| y | 8 | Y | 28.61 | 3.88-4.5 | 4.09 (0.25) | 1441-17738 | 1647.8 (107.7) | *Caryophyllia* sp. 2, Porifera encrusting sp. 28,  *Lepidisis* sp.. *Psolus squamatus*,  *Parantipathes* sp.1 |
| z | 10 | Y | 27.89 | 4.4-4.65 | 4.55 (0.07) | 1523-1567 | 1556.5 (13.8) | *Solenosmilia variabilis*, Porifera encrusting sp. 6,  *Keratoisis* sp. 2,  *Caryophyllia* sp. 2, Ophiuroidea sp. 4 |
| aa | 24 | Y | 28.14 | 4.3-5.5 | 4.67 (0.39) | 1315-1607 | 1462.2 (77.1) | *Solenosmilia variabilis*,  *Ophiactis balli*,  *Solenosmilia variabilis* (dead structure), Isididae sp. 2, Zoanthidae sp.6, Porifera encrusting sp. 6,  *Anthomastus grandiflora* |
